# CD4^+^c-Met^+^Itgα4^+^ T cell subset promotes murine neuroinflammation

**DOI:** 10.1186/s12974-022-02461-7

**Published:** 2022-04-29

**Authors:** Mahdia Benkhoucha, Ngoc Lan Tran, Gautier Breville, Isis Senoner, Paul F. Bradfield, Thalia Papayannopoulou, Doron Merkler, Thomas Korn, Patrice H. Lalive

**Affiliations:** 1 Department of Pathology and Immunology, Faculty of Medicine, University of Geneva, Geneva, Switzerland; 2 Department of Neurosciences, Division of Neurology, University Hospital of Geneva, Geneva, Switzerland; 3 MesenFlow Technologies SARL, Chemin des Aulx 14, Geneva, Switzerland; 4 Division of Hematology, Department of Medicine, University of Washington, Seattle, WA USA; 5 Division of Clinical Pathology, Department of Pathology and Immunology, Faculty of Medicine, University of Geneva, Geneva, Switzerland; 6 Department of Neurology, Klinikum rechts der Isar, Technical University of Munich, Munich, Germany; 7 Institute for Experimental Neuroimmunology, Klinikum rechts der Isar, Technical University of Munich, Munich, Germany; 8 Munich Cluster for Systems Neurology, SyNergy, Munich, Germany

**Keywords:** c-Met, HGF, T lymphocytes, Integrin, Transmigration, Neuroinflammation, EAE, MS

## Abstract

**Objective:**

c-Met, a tyrosine kinase receptor, is the unique receptor for hepatocyte growth factor (HGF). The HGF/c-Met axis is reported to modulate cell migration, maturation, cytokine production, and antigen presentation. Here, we report that CD4^+^c-Met^+^ T cells are detected at increased levels in experimental autoimmune encephalomyelitis (EAE), a mouse model of multiple sclerosis (MS).

**Methods:**

c-Met expression by CD4^+^ T cells was analyzed mostly by flow cytometry and by immunohistochemistry from mice and human PBMCs. The in vivo role of CD4^+^c-Met^+^ T cells was assessed in EAE.

**Results:**

CD4^+^c-Met^+^ T cells found in the CNS during EAE peak disease are characterized by a pro-inflammatory phenotype skewed towards a Th1 and Th17 polarization, with enhanced adhesion and transmigration capacities correlating with increased expression of integrin α4 (Itgα4). The adoptive transfer of Itgα4-expressing CD4^+^Vα3.2^+^c-Met^+^ T cells induces increased disease severity compared to CD4^+^Vα3.2^+^c-Met^−^ T cells. Finally, CD4^+^c-Met^+^ T cells are detected in the brain of MS patients, as well as in the blood with a higher level of Itgα4. These results highlight c-Met as an immune marker of highly pathogenic pro-inflammatory and pro-migratory CD4^+^ T lymphocytes associated with neuroinflammation.

**Supplementary Information:**

The online version contains supplementary material available at 10.1186/s12974-022-02461-7.

## Background

Multiple sclerosis (MS) is an immune-mediated inflammatory demyelinating disease of the central nervous system (CNS) [1, 2]. Numerous immune mediators have been detected within MS lesions, including CD4^+^ and CD8^+^ T lymphocytes, suggesting their participation in MS pathogenesis [2, 3]. Autoreactive CD4^+^ T cells, including IFNγ-secreting T helper (Th) 1, IFNγ/IL‐17-secreting Th17, or IFNγ/GM‐CSF-secreting cells [2, 4–6], play an important role in MS, which is supported by data from the experimental autoimmune encephalomyelitis (EAE) mouse models [7].

In MS, brain-reactive T cells invade the CNS and induce a self-destructive inflammatory process. Although the exact antigen targeted by these cells are unknown, it is supposed that autoinflammatory T cells recognized a specific antigen expressed by oligodendrocytes at the surface myelin sheath [8]. T cell infiltrates are found within the parenchyma, meninges and in the cerebrospinal fluid (CSF) [9]. Homing of CD4^+^ T cells from the periphery into the CNS during MS and EAE involves specific adhesion molecules including integrin alpha 4 (Itgα4), one of the subunit of very late antigen 4 (VLA4) integrin [10]. Based on this observation, monoclonal antibodies targeting Itgα4 have been developed. In mice, they prevent the development of EAE [10] and in MS patients, natalizumab is used as a disease modifying therapy [11], and is considered as one of the most potent drug to treat the disease. It has been shown that Itgα4 blockade does not uniformly block lymphocyte homing and function [12, 13]. Indeed, conditional deletion of Itgα4 on T cells leads to a Th17-mediated form of EAE, because Itgα4 is specifically required for the homing of Th1 but not Th17 cells into the CNS [12]. During EAE, both Itgα4 and P-selectin glycoprotein ligand (PSGL-1) are shown to mediate the rolling of endogenous leukocytes in inflamed superficial brain vessels [14]. Moreover, T cell crawling on the blood–brain barrier (BBB) against the blood flow to find a permissive site for diapedesis is mediated by a high affinity leukocyte function-associated antigen (LFA)-1 molecule [15]. In addition to the role of integrins, T cells can invade the CNS under the influence of chemokine-dependent migration through the BBB [16]. Both in human and mice, studies have found that specific chemokine receptors are expressed by Th cell subsets. CCR5 and CXCR3 are preferentially expressed on Th1 cells, while Th17 cells express chemokine receptors, such as CCR2, CCR4 and CCR6 [17, 18].

c-Met is a transmembrane tyrosine kinase receptor that is the unique receptor identified for hepatocyte growth factor (HGF). HGF/c-Met axis is known to be involved in cell survival, cell growth and regeneration [19, 20]. HGF/c-Met axis also modulates several inflammatory-mediated diseases by acting on a wide variety of cells [21], including in EAE [22, 23]. We have shown recently that HGF modulates c-Met-expressing CD8^+^ T cell function ex vivo in MOG_35–55_-induced EAE [24]. In addition, we recently identified the presence of human c-Met-expressing CD4^+^ T cells upon T cell receptor (TCR) triggering. Phenotypic and functional analyses of CD4^+^c-Met^+^ T cells revealed an enhanced pro-inflammatory phenotype skewed towards a Th17 and Th17.1 polarization, with increased production of IL-17 alone or both IL-17/IFNγ (double positive) and higher levels of Itgα4 compared to CD4^+^c-Met^−^ T cells [25].

While most data has highlighted a protective role of HGF in inflammatory and autoimmune animal models upon immunomodulation of dendritic cells [22, 26, 27], little is known about the contribution of the HGF/c-Met axis in regulating T cell development and functions. A previous report has suggested that the HGF/c-Met axis may control T cell development in the thymus. Indeed, analysis of c-Met expression in the mouse thymus revealed that c-Met mRNA transcripts are detected at higher levels during early ontogenesis than in adult thymus [28]. In a related model, HGF was found to induce a “heart-homing” signature to T cells during their activation [29]. Finally, and most importantly, we established that HGF directly inhibited the effector and antitumor activities of a subpopulation of c-Met-expressing cytotoxic T lymphocytes (CTLs) [30].

In the present study, we investigated the role of c-Met-expressing CD4^+^ T cells using a series of MOG_35–55_ T cell-mediated EAE and adoptive transfer models. We found that a subpopulation of effector CD4^+^ T cells expressed c-Met in EAE, with a predominance at peak disease. These cells were transcriptomically distinct from conventional CD4^+^c-Met^−^ T cells, expressed high level of Itgα4 (a subunit of VLA4 integrin), chemokine receptors and homing molecules, and had the capacity to migrate to inflammation sites. Our results offer an ex vivo and in vitro phenotypic and functional characterization of this population and open new considerations on CD4^+^c-Met^+^ T cells as a highly pathogenic T cells subpopulation that contributes significantly to EAE development. Finally, CD4^+^c-Met^+^ T cells can be detected in the brain of MS patients and expressed higher levels of Itgα4 in the blood of MS patients compared to controls. Our findings suggest that c-Met expression by CD4^+^ Th1 cells confers specific pro-inflammatory and migratory properties, related to Itgα4 expression.

## Methods

### Standard protocol approvals

Venous blood was collected from seven untreated MS patients (3 males and 4 females, mean age 38 years) and eight patients with other neurological diseases (OND) (4 males and 4 females, mean age 46 years, cephalalgia n = 3, epileptic seizure (idiopathic) n = 1, functional neurological disorder n = 1, cognitive impairment n = 3). PBMCs were isolated and immediately used or cryopreserved in accordance with the ethical committee of Geneva Hospital (Switzerland).

For histopathological analysis, brain sections derived from biopsy of MS lesion (female, 42 years, late active relapsing–remitting MS) was obtained from the Hôpitaux Universitaires de Genève (HUG). Its use for scientific purposes was in accordance with institutional ethical guidelines and approved by the ethics committee of the University of Geneva (Switzerland). Informed consent was obtained from the subject.

### Mice

C57BL/6J (H-2^b^) female mice and MOG_35–55_-specific TCR transgenic (2D2) mice derived in C57BL/6J background [31] were purchased from Charles River (France). 2D2 CD4^Cre^Itgα4^fl/fl^ mice were provided by Pr. Tomas Korn (Technische Universität München), MTA from Dr. Papayannopoulou was obtained for Itgα4^fl/fl^ mice [32]. Animals were housed in a specific pathogen-free barrier facility at the Medical Center of Geneva, Faculty of Medicine (Geneva, Switzerland). Animal care and all experimental protocols and procedures were reviewed and approved by the Institutional Animal Care and Use Committee of the Geneva University School of Medicine.

### Induction and assessment of EAE

Mice were immunized subcutaneously in the flank with 200 μg MOG_35–55_ peptide (MEVGWYRSPFSRVVHLYRNGK; Anawa Trading) in complete CFA (DIFCO Laboratories), and 300 ng of pertussis toxin (PTX, Sigma-Aldrich) in phosphate-buffered saline (PBS) was administered intravenously (i.v.) on days 0 and 2. Individual animals were observed daily, and clinical scores were assessed with a 0–5 point scoring system as follows: 0 = no clinical disease, 1 = loss of tail tone only, 2 = mild monoparesis or paraparesis, 3 = severe paraparesis, 4 = paraplegia and/or quadraparesis and 5 = moribund or death. Mice exceeding 2 days with a score of 3,5 were euthanized immediately. For the adoptive transfer, polarized MOG_35–55_-specific Th1 cells was performed as described by Yang et al. [33]. Briefly, splenocytes from either control 2D2 Vα3.2^+^CD4^Cre^ mice or 2D2 Vα3.2^+^CD4^Cre^Itgα4^fl/fl^ were cultured and stimulated with MOG_35–55_ (20 μg/ml) in the presence of 5 μg/ml of IL-2/IL-7 (Biolegend) for 2 days. Then, cells were expanded with IL-2/IL-7 for another 4 days. Subsequently, cells were reactivated for 24 h with plate-bound anti-CD3 and anti-CD28 (1 μg/ml) in the presence of IL-12 (20 ng/ml; Biolegend) and IL-18 (20 ng/ml; Biolegend). Activated T cells were collected, sorted by flow cytometry for c-Met^−^ or c-Met^+^, washed, and 3 × 10^6^ cells were transferred intraperitoneally (i.p.) into recipient mice. PTX (67 ng/mouse) was injected i.v. at days 0 and 2.

### Preparation and treatment of splenic 2D2 Th1 T cells in vitro

Th1 polarized cells were obtained after optimization of protocols described previously in [33]. Briefly, 4 × 10^6^ 2D2 total erythrocyte-lysed spleen cells per well were cultured for 2 days in the presence of 20 µg/ml recombinant MOG_35–55_ (rMOG; ANAWA) in 12 well-plates in complete RPMI-1640 containing 10% fetal calf serum (FCS), 25 mM HEPES, 2 mM l-glutamine, 50 U/ml penicillin, and 50 μg/ml streptomycin. The following cytokines and antibodies were further added in the culture for another 4 days: IL-2/IL-7 (5 μg/ml; Biolegend), IL-12 (20 ng/ml; Biolegend), IL-18 (20 ng/ml; Biolegend) and anti-IL-4 (400 ng/ml; Thermo Fisher Scientific).

After 6 days of polarization, 2D2 Th1 cells were harvested, stained at 4 °C for 30 min with anti-CD4, anti-Vα3.2 and anti-c-Met antibodies (Abs), and sorted by flow cytometry in CD4^+^ Vα3.2^+^c-Met^−^ and CD4^+^ Vα3.2^+^c-Met^+^ cells using FACS Aria SORP II cell sorter (BD Biosciences). Live cells were gated based on their morphology (forward scatter vs side scatter) and by DAPI dye exclusion. Sorted T cells were incubated with irradiated syngeneic DCs loaded or not with 20 µg/ml of MOG_35–55_ for 48 h.

### CNS cell isolation

Central nervous system (CNS) mononuclear cells were isolated from mice after cardiac perfusion with PBS. Briefly, minced CNS (brain and spinal cord) tissues were digested with collagenase D (2.5 mg/ml; Roche Diagnostics) at 37 °C for 60 min. Mononuclear cells were isolated by discontinuous Percoll gradient (70/30%; Sigma-Aldrich) centrifugation. Lymphocytes were collected from the 30:70% interface and washed. Total cell numbers were determined by counting on a hemocytometer, and viability was assessed by Trypan blue exclusion.

### Human PBMC isolation

PBMCs from MS and OND patients were obtained by density gradient centrifugation of human peripheral blood over Ficoll–Paque™ PLUS (GE Healthcare, Life Science). Isolated PBMCs were resuspended in FACS buffer (DPBS free calcium and magnesium, and supplemented with 0.5% bovine serum albumin) for flow cytometric analysis.

### Flow cytometric analysis

Single-cell suspensions from spleens, lymph nodes, and CNS from d0, d14 and d21, and also cells generated in vitro were incubated in blocking solution (PBS with 1% of fetal calf serum) for 20 min on ice prior the staining to block non-specific Fc-mediated interactions. Then they were stained for 30 min at 4 °C with appropriate fluorochrome-conjugated Abs (1:100) (Additional file 1: Table S1) or isotype-matched irrelevant Abs to determine background fluorescence. For intracellular cytokines and molecular staining of IFNγ, TNFα, IL-17 and GM-CSF, T cells were stimulated with phorbol myristate acetate (PMA, 50 ng/ml) plus ionomycin (500 ng/ml) in the presence of brefeldin A (10 μg/ml; Sigma-Aldrich) and then fixed and permeabilized using BD Cytofix/Cytoperm Plus Kit (BD Biosciences). Samples were processed on a FACS Gallios or LSRFortessa flow cytometer (BD Biosciences) and analyzed using FlowJo analysis software (version 10.3). Live, apoptotic, and dead populations were defined on the basis of 7-AAD Viability Staining Solution from eBioscience according to the manufacturer’s instructions. To determine the viability of cells prior to the fixation and permeabilization, we used the LIVE/DEAD™ Fixable Aqua Dead Cell Stain Kit (Thermo Fisher Scientific).

Human Fc-block (BD Pharmingen) was used to block non-specific binding of Fc-receptors prior to the extracellular staining of human PBMCs. They were then stained for 30 min at 4 °C with appropriate fluorochrome-conjugated Abs (1:100) (Additional file 1: Table S1) or isotype-matched irrelevant Abs to determine background fluorescence. To exclude dead cells, samples were co-stained with DRAQ7 (Invitrogen) and analyzed using a LSRFortessa™ flow cytometer (BD Bioscience) with standard equipment.

### Measurement of effector molecules and cytokines

IFNγ, TNFα, CCL3, CXCL2 and CXCL10 levels were detected in culture supernatants from the coculture of 2D2 polarized Th1 (c-Met^−^ vs c-Met^+^) and irradiated DCs loaded or not with MOG_35__–__55_ peptide using V-PLEX Mouse Cytokine 29-Plex Kit from MSD company according to the manufacturer’s instructions.

### Immunofluorescence

Cytospin splenocytes, lymph nodes (LN) and CNS infiltrating cells from peak disease of EAE mice (d14) were fixed with acetone. Slides were blocked for 30 min in PBS with 1% bovine serum albumin (BSA). Abs against mouse c-Met (clone EP1454Y; Abcam) and CD4 (clone GK1.5; Thermo Fisher Scientific) were applied at 1:50 and 1:100, respectively. Sections were incubated overnight with both Abs. After several washes with PBS, secondary antibody against Rabbit (Invitrogen) at 1:100 was applied for 1 h at room temperature. Slides were mounted with ProLong Gold anti-fade reagent with DAPI (Life Technologies) and kept at 4 °C. The slides were imaged in the Geneva University School of Medicine bioimaging core facility using a Leica SP5 Axiocam system (Leica MicroImaging).

The frontal subcortical white matter part of the human brain biopsy from MS patient was prepared in 10% neutral buffer formalin overnight and embedded in paraffin as described previously [34]. The antigen retrieval using a Marmite Pascal (Citrate buffer pH 6.0, 125° for 30 s) was performed to avoid unspecific binding. Human section was blocked 15 min in PBS with 10% FCS. Abs against c-Met (clone EP1454Y; Abcam) and CD4 (clone 4B12; Invitrogen) were applied at 1:50 and 1:10, respectively, in Dako Diluent (Dako). Section were incubated overnight at 4 °C with primary antibodies. After several washes with Wash Buffer (Dako), secondary antibodies against rabbit (Jackson ImmunoResearch) and mouse (Life Technologies) at 1:200 in PBS containing Dapi (Life Technologies at 1:2000) was applied for 1 h at room temperature. The slide was mounted with a coverslip and scanned using the Pannoramic 250 FLASH II (3DHISTECH) Digital Slide Scanner at × 20 magnification.

### Western blotting

Purified CD4^+^ T cells were homogenized using a polytron in lysis buffer (50 mM Tris–HCl [pH 7.5], 250 mM NaCl, 1% Triton X‐100, 1 mM EDTA, and 1 mM DTT) containing complete protease inhibitors (Roche). Equal amounts (20 μg) of total protein from each sample were transferred to 8% sodium dodecyl sulfate (SDS)-polyacrylamide gel and blotted onto an Immobilon‐P polyvinylidene difluoride (PVDF) membrane (Millipore). The level of c‐Met proteins was detected using diluted (1:100) rabbit monoclonal anti‐c-Met Ab (clone EP1454Y; Abcam), followed by a peroxidase‐conjugated secondary Ab to rabbit IgG1 (Bio-Rad), and then visualized using chemiluminescence (Supersignal; Pierce). The blot was also probed with mouse monoclonal anti‐β‐actin (clone 15G5A11/E2; Sigma-Aldrich) as a loading control.

### RNA isolation and real-time quantitative PCR

RNA was prepared from MACS-sorted splenic CD4^+^ T cells at day 0 and day 6 post 2D2 Th1 polarization followed by separation with FACS Aria on CD4^+^Vα3.2^+^c-Met^−^ or CD4^+^Vα3.2^+^c-Met^+^. Total RNA extractions were performed with the Qiagen RNeasy Mini Kit and subjected to DNase I (Roche Diagnostics) digestion. Random hexamer primers (Promega, Madison, WI) and Superscript II RNase H reverse transcriptase (Invitrogen, Carlsbad, CA) were used to generate cDNA. All transcripts were quantified by real-time PCR analysis using SYBR Green as the detection agent. The PCR was performed with the 7500 Real-Time PCR System (Applied Biosystems). The following specific primers for the target genes were obtained from Microsynth: *c‐Met* (forward: CCAGAGCCACATGCTCCTAGA; reverse: AGCTGGTCCTTTGTTTGAAAGAA). Real‐time PCR was carried out using SYBR Green PCR Core Reagents (Applied Biosystems). The PCR conditions were 50 °C for 2 min, 95 °C for 10 min, and 45 cycles of 95 °C for 15 s, 60 °C for 1 min, and 72 °C for 30 s. The expression level of the housekeeping gene *β‐actin* (actb) (forward: CTAAGGCCAACCGTGAAAAGAT; reverse: CACAGCCTGGATGGCTACGT) was used for normalization. Expression levels of mRNA were analyzed with SDS 2.1 (Applied Biosystems) and quantified using the relative standard curve method, followed by comparison with the results from control samples. For all reactions, each condition was performed in triplicate, and each experiment was repeated at least three times.

### Microarray assay

1 ng of total RNA were used as input for the preparation of labeled ds-cDNA using the GeneChip Pico Reagent kit (Thermo Fisher Scientific). 9 cycles of pre-IVT amplification were performed according to recommendations. Targets were then hybridized on Human Clariom S arrays. Hybridization, scanning, and raw data processing were performed at the Genomics Core Facility, Geneva University, Switzerland.

### Cell adhesion assay

Mice brain derived endothelial cell line (bEnd.3) were cultured to confluence on 48 wells plate, bEnd.3 cells were treated or not with TNFα (10 ng/ml) overnight. 2D2 CD4^+^Vα3.2^+^c-Met^−^ and 2D2 CD4^+^Vα3.2^+^c-Met^+^ Th1 cells were labeled with 5 μM carboxyfluorescein diacetate succinimidyl ester (CFSE; Thermo Fisher Scientific) according to the manufacturer’s instructions and then incubated with bEnd.3 cells on the chamber slides for 1 h at 37 °C. After incubation, the unbound Th1 cells were rinsed away three times, and the number of T cells that firmly bound to bEnd.3 cells was counted in three fields by fluorescent microscopy. CellProfiler™ software was used for the cell counting.

### Transwell assay

Transwell migration assays were performed to assess 2D2 CD4^+^Vα3.2^+^c-Met^−^ and CD4^+^Vα3.2^+^c-Met^+^ cell migration in presence of CXCL12 (100 ng/ml) in the lower chamber. bEnd.3 endothelial cells were coated on the transwell chamber (24-well insert, diameter: 6.5 mm, pore size: 5 µm, Corning, Vitaris AG) and activated or not (control) with TNFα (10 ng/ml). After 6 days of polarization, 10^5^ sorted 2D2 CD4^+^Vα3.2^+^c-Met^−^ or CD4^+^Vα3.2^+^c-Met^+^ T cells were treated with 10 ug/ml of either anti-Itgα4 or anti-ItgαL or both antibodies for 1 h, then washed, plated in the top chamber and incubated for 6 h at 37 °C to allow the cells to attach and transmigrate. The cells contained in the lower chamber are harvested and the relative number of transmigrated cells was determined using fluorescent count-bead (Thermo Fisher Scientific) by flow cytometer to normalize the number of transmigrated cells.

### Shear flow assay (MesenFlow Technologies)

Human umbilical vein endothelial cells (HUVECs) were cultured in chamber slides for 2–3 days and then treated for a further 24 h using a chronic activation protocol [35]. Briefly, the first stage of activation consisted of an overnight stimulation with TNFα (1000 U/ml) and IFNγ (500 U/ml). T cells isolated from 2D2 transgenic mice were collected form the spleen and expanded in vitro to generate Th1 cells. The flow assay setup contained a heated microscope chamber (37 °C) and a calibrated pump, where flow was generated over HUVEC monolayers by perfusing wash buffer (M199 culture media, 0.1% BSA) with or without T cell suspension. The flow rate was representative of small venules/capillaries (0.05–0.1 Pa). Assays were initiated with a 10 min wash phase to remove culture media, followed by a second-stage of wash, where CXCL12 (0.5 μM) was perfused over the monolayer for 5 min and stasis for 15 min for endothelium activation. Wash buffer was then pumped over the HUVECs for 10 min to remove any unbound CXCL12. Suspensions of T cells were then perfused over the HUVECs for 6 min followed by 50 min of wash buffer. Images of the captured T cells were made using phase contrast microscopy and a camera. Individual images were recorded every 30 s and compiled into time-lapse short movie sequences, allowing analysis of individual T cells over large areas. T cells adherent to the surface of the HUVECs had a phase-white appearance, whereas those that have transmigrated have a phase-black appearance. Adhesion events were recorded as the total of number of cells per unit field (mm^2^). Transmigration events were presented as a percentage of total T cells captured from flow per unit field, and the total number of transmigrated T cells per unit field (mm^2^). All experiments were carried out using triplicate fields.

### Statistical analysis

Statistical calculations were performed using the statistical analysis software GraphPad Prism, version 8.0. Data were expressed as mean values ± standard error mean (SEM), and the differences between groups were evaluated by unpaired Student’s *t* test or Wilcoxon matched-pairs signed rank test. Intergroup comparisons were conducted by two-way analysis of variance (ANOVA) followed by Tukey’s post hoc test to determine significant differences between experimental groups. *p* values < 0.05 were considered statistically significant. If not mentioned, differences are not statistically significant.

## Results

### CD4^+^c-Met^+^ T cells population is increased in MOG_35–55_-induced EAE at peak disease and presents a pro-inflammatory profile

**Fig. 1:**
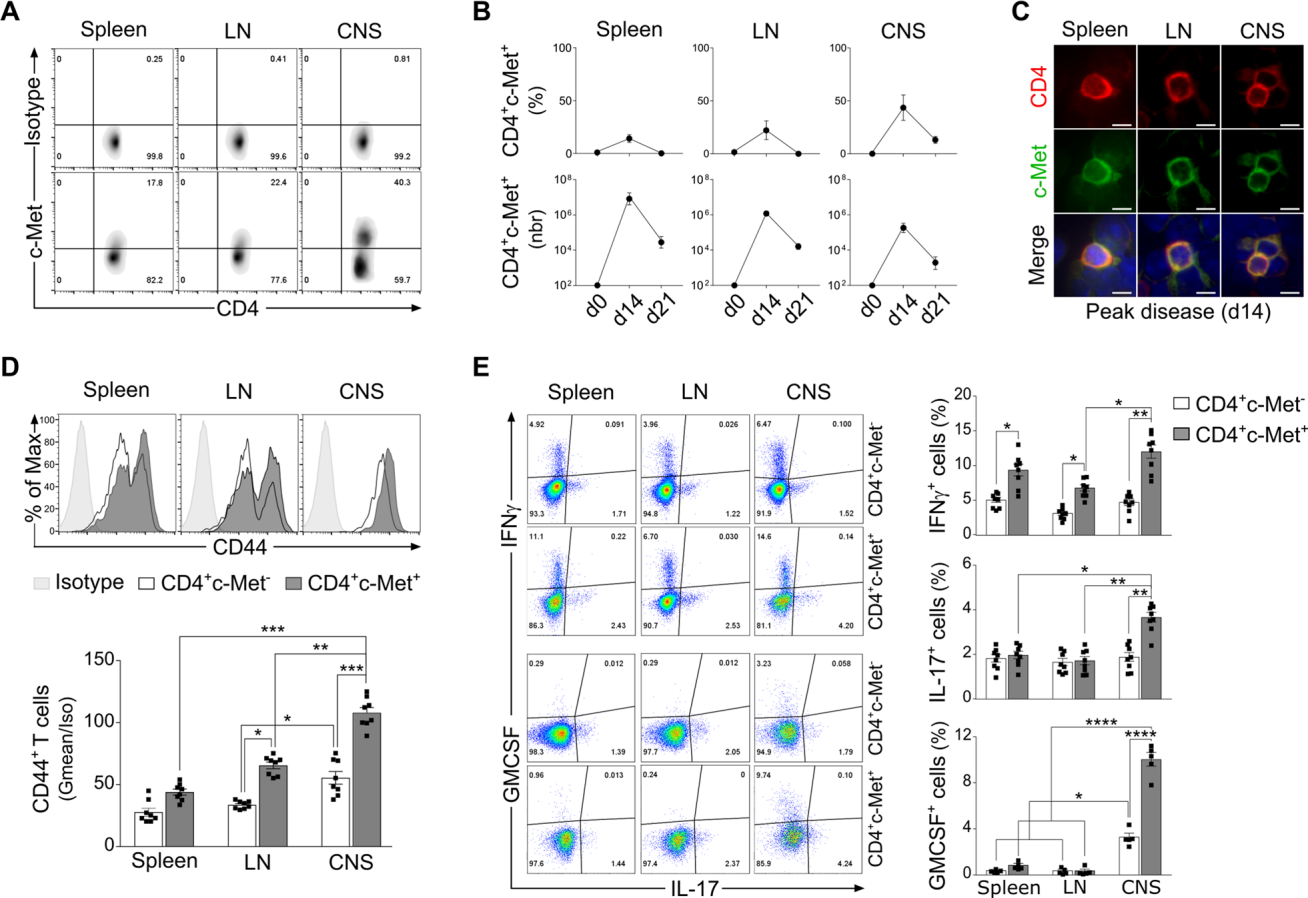
CD4^+^c-Met^+^ T cells are increased in MOG_35-55_-induced EAE at peak disease and exert a pro-inflammatory profile. **A** Representative flow cytometric density plots for the identification of c-Met-expressing CD4^+^ T cells in MOG_35–55_-induced EAE at peak disease (d14), extracted from the spleen, lymph nodes (LN) and central nervous system (CNS). Live CD4^+^ T lymphocytes were defined as 7AAD^−^CD45^+^CD3^+^CD4^+^ (not shown). Percentage of CD4^+^c-Met^+^ population were indicated in the density plot quadrants (CD4 vs c-Met) at d14. c-Met isotype control antibody (top panels) was used to define c-Met expression on CD4^+^ T cells in the three compartments (bottom panels). **B** Flow cytometric quantification of CD4^+^c-Met^+^ T cells in spleen, LN and CNS during different EAE phases: preimmunization (d0), peak disease (d14) and chronic phase (d21). CD4^+^c-Met^+^ T cells are gated as described in (A). The frequency (top panel) and absolute number (lower panel) of CD4^+^c-Met^+^ T cells are shown in indicated organs and indicated timepoints. **C** Representative double immunofluorescent staining images for CD4 (red) and c‐Met (green) of cytospin spleen, LN and CNS cells at peak disease (d14). Cell nuclei were counterstained with DAPI (blue). Scale bar = 10 μm. Similar results were obtained with three other mice. **D** Representative flow cytometric histograms (top panels) and geometric mean fluorescence intensity (Gmean/Isotype) quantification (lower panel) of CD44 expression on CD4^+^c-Met^−^ and c-Met^+^ T cells in spleen, LN and CNS. **E** Representative flow cytometric plots (lefts panels) for the identification of IFNγ, GM-CSF and IL-17 producing CD4^+^c-Met^−^ and c-Met^+^ T cells isolated at peak disease (d14) from the spleen, LN and CNS. Cells were stimulated in vitro with PMA/Ionomycin for 4 h, incubated with Brefeldin A, and stained for extracellular markers and intracellular cytokines. Percentage (right panels) of IFNγ, GM-CSF and IL-17 positive CD4^+^c-Met^−^ and c-Met^+^ T cells are depicted. Mean values ± SEM for n = 8 mice/group are shown; *p ≤ 0.05, **p ≤ 0.01, ***p ≤ 0.001, ****p ≤ 0.0001 by two-way ANOVA followed by Tukey’s post hoc test

We first assessed the expression of the HGF receptor c-Met during the course of MOG_35__–__55_-induced EAE. Mice were monitored for up to 21 days (recovery phase) post-immunization (Additional file 1: Fig. S1A). As shown in Additional file 1: Fig. S1B, immunized mice started to show EAE disease signs 7 day post-EAE induction and reached the highest incidence rate in 12 days. No expression of c-Met was detected in the pre-immunization phase (d0) within CD45^+^CD3^+^CD4^+^ expressing T cells, while these cells expressed significant levels of c-Met receptor in the spleen, lymph node (LN) and CNS at peak disease (d14), in percentage (as gated in Fig. 1A) and cell number (Fig. 1B). By immunofluorescence, we confirmed c‐Met expression in CD4^+^ T cells from spleen, LN and CNS at peak disease (d14) (Fig. 1C). c‐Met expressing CD4^+^ T cells population decreased at the chronic phase (d21) in all compartments (Fig. 1B).

When examining the expression of T cell activation marker CD44 at peak disease (d14), we found that the level of activation of CD4^+^c-Met^+^ T cell, compared to their CD4^+^c-Met^−^ counterpart, were increased in spleen, LN and CNS, with a maximum detected in the CNS (Fig. 1D). We then assessed the proportions of Th1 and Th17 infiltrated T cells in spleen, LN and CNS at peak disease, and we showed by intracellular flow cytometry that CD4^+^c-Met^+^ T cells, purified from the three compartments, expressed higher level of IFNγ compared to their CD4^+^c-Met^−^ T cells counterpart (Fig. 1E, top panels). Moreover, a larger proportion of CD4^+^c-Met^+^ T cells producing IL-17 and GM-CSF were detected specifically in the CNS, when compared to the LN and spleen. No difference of IL-17 and GM-CSF production was observed in the periphery (LN and spleen) (Fig. 1E, bottom panels). No difference was observed in TNFα production at peak disease in both populations (Additional file 1: Fig. S1C).

### CD4^+^c‐Met^+^ T cells expressed higher level of Itgα4 and chemokine receptors in the CNS of MOG_35–55_-induced EAE at peak disease

**Fig. 2:**
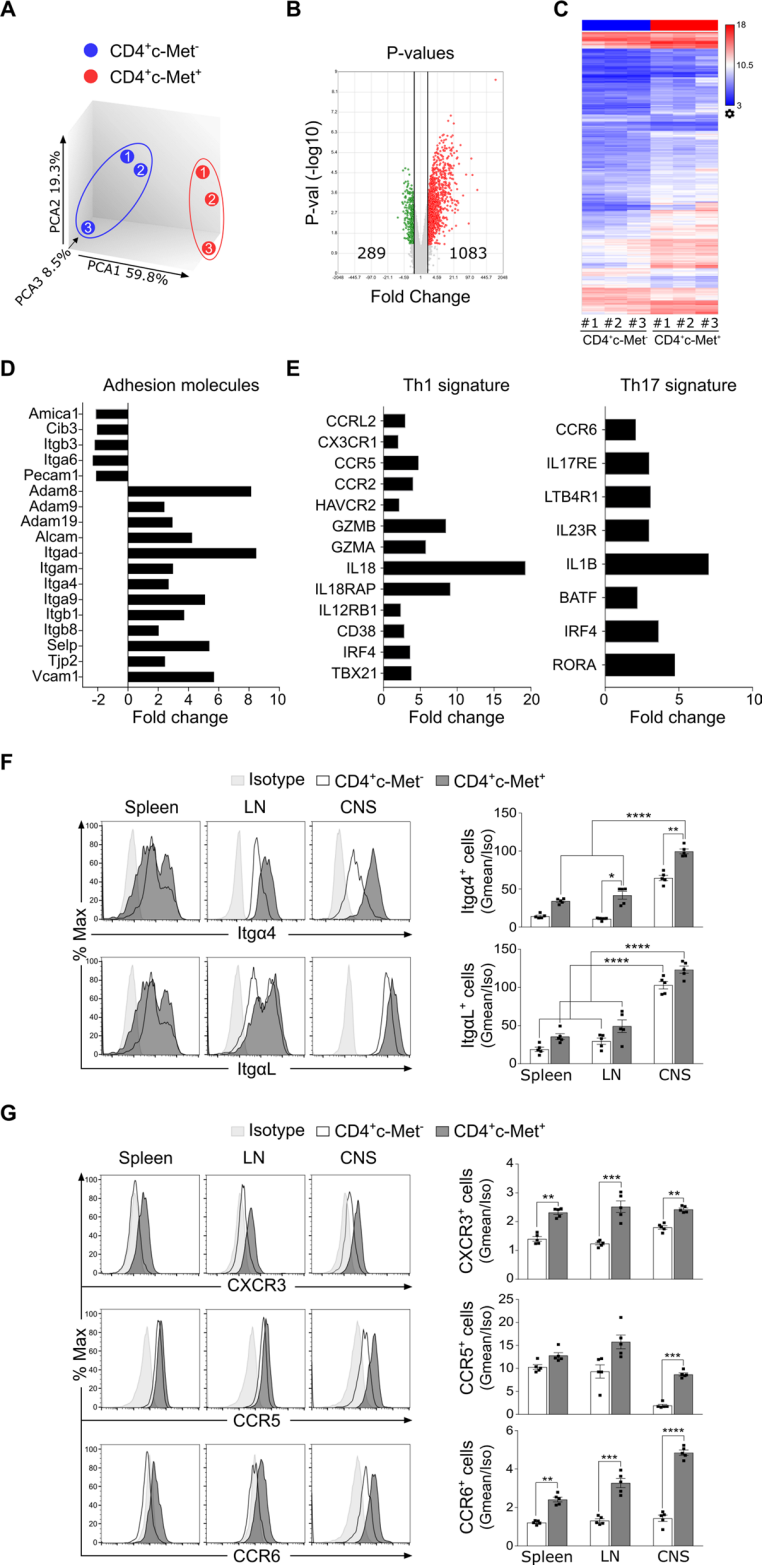
CD4^+^c‐Met^+^ T cells expressed higher level of Itgα4 and chemokine receptors in MOG_35-55_-induced EAE at peak disease. **A** Heat map analysis of microarray data showing differentially expressed genes of CD4^+^c-Met^−^ and c-Met^+^ T cells isolated from CNS at EAE peak disease (d14) mice. The global gene expression profiles were analyzed by Principal Component Analysis (PCA). The first three principal components of microarray analysis data (PC1, PC2 and PC3) in X, Y and Z, respectively, are shown, and demonstrated the expression profile of three samples/groups (CD4^+^c-Met^−^ blue#1–3; CD4^+^c-Met^+^ Red#1–3). **B** Volcano plots comparing genes expressed by CD4^+^c-Met^−^ and c-Met^+^, showed a total of 1372 differentially regulated genes (1083 up-regulated, red, and 289 down-regulated, green) with at least twofold changes by *P* value (y axis) and fold change (x axis). **C** Heat map analysis of microarray data showing hierarchical clustering of differentially expressed probes between CD4^+^c-Met^−^ and CD4^+^c-Met^+^ cell population (three samples per group). Red and blue colors indicate differentially up- or down-regulated genes, respectively. The mean signals were background corrected and transformed to the log2 scale. Gene comparisons were done for CD4^+^c-Met^+^ and CD4^+^c-Met^−^ with at least twofold changes and p < 0.05 at the 95% confidence level were considered as significant. **D** KEGG pathways analysis revealed that selected genes were involved in several key pathways related to adhesion molecules associated with regulation of EAE development under an inflammatory microenvironment. Gene expression profiles are represented in fold change by CD4^+^Vα3.2^+^c-Met^+^ cells compared to CD4^+^Vα3.2^+^c-Met^−^ cells. **E** KEGG pathways analysis revealed that selected genes involved in Th1 (left panel) or Th17 (right panel) were upregulated in CD4^+^Vα3.2^+^c-Met^+^ cells when compared to CD4^+^Vα3.2^+^c-Met^−^ cells. Gene expression profiles are represented in fold change by CD4^+^Vα3.2^+^c-Met^+^ cells compared to CD4^+^Vα3.2^+^c-Met^−^ cells. **F** Representative flow cytometric histograms (left panels) and Gmean/isotype quantifications (right panels) of integrins α4 (Itgα4, VLA4 subunit) and αL (ItgαL, LFA1 subunit) expression by CD4^+^c-Met^−^ and c-Met^+^ T cells from spleen, LN and CNS at peak disease (d14). **G** Representative flow cytometric histograms (left panels) for chemokine receptors expression, such as CXCR3, CCR5 and CCR6 in CD4^+^c-Met^−^ and c-Met^+^ T cells isolated from spleen, LN and CNS at peak disease (d14). Gmean/isotype quantifications (right panels) representing mean values ± SEM for n = 5 mice/group are shown; *p ≤ 0.05, **p ≤ 0.01, ***p ≤ 0.001, ****p ≤ 0.0001 by two-way ANOVA followed by Tukey’s post hoc test

CD4^+^ T cells isolated from the CNS in MOG_35–55_-induced EAE animals were assessed by Affymetrix Microarrays at peak disease. The correlation between the biological triplicates in each group was calculated using principal component analysis (PCA) mapping as shown in the three-dimensional graphic (the distance between two plotted points is proportional to the degree of similarity between the gene expression profiles). The PCA plot represents a projection on the first three principal components. Here, we found that a total variance of 87.6% (sum of 59.8%, 19.3%, and 8.5%) confirmed that CD4^+^c-Met^+^ T cell samples clustered apart from samples belonging to the CD4^+^c-Met^−^ T cell group (Fig. 2A). The analysis of microarray data had identified 1372 probe sets that are differentially expressed based on the criteria of a fold change of ≥ 2 and adjusted p < 0.05 between CD4^+^c-Met^+^ and CD4^+^c-Met^−^. We detected 289 down-regulated and 1083 up-regulated genes as shown in the volcano plot (Fig. 2B) and in the heat map presentation (Fig. 2C). To determine which pathway might be characteristic for CD4^+^c-Met^+^ compared to CD4^+^c-Met^−^ T cells population, KEGG pathway analysis was used to authenticate pathways and understand biological functions of significantly differentially expressed genes (DEGs). The results indicated that the up-regulated DEGs were enriched in several pathways, such as the adhesion molecules (Fig. 2D), and in Th1 and Th17 signature (including master regulator genes, chemokine receptors, cytokines, cytokine regulators, and cytokine receptors, Fig. 2E).

The migration of activated T cells into the CNS is mediated by adhesion molecules [15] and directed by chemokines [36]. Based on microarray data results, we analyzed the expression of integrins, including Itgα4 (VLA4 subunit), ItgαL (LFA-1 subunit) and P-selectin glycoprotein ligand-1 (PSGL-1). These adhesion molecules were selected, because they are known to be predominantly involved in T cells trafficking across the blood brain barrier (BBB) during EAE [37, 38]. Flow cytometric analysis at EAE peak disease on CD4^+^ T cells extracted from the spleen, LN and CNS showed different expression profile of adhesions molecules and chemokines receptors between CD4^+^c-Met^+^ and CD4^+^c-Met^−^ T cell populations. While the expression of Itgα4 and ItgαL are both upregulated in the CNS compared to the other compartments, only Itgα4 is overexpressed in CD4^+^c-Met^+^ population compared to CD4^+^c-Met^−^ cells, in both LN and the CNS (Fig. 2F). By contrast, no difference was observed in PSGL-1 expression (Additional file 1: Fig. S1D). These results suggest an increased pro-migratory profile of CD4^+^c-Met^+^ population compared to CD4^+^c-Met^−^ T cells through an overexpression of Itgα4.

We next analyzed the levels of selected Th1 chemokine receptors, such as CCR5 and CXCR3, and the level of the Th17 chemokine receptors, such as CCR6. These chemokines showed a significant multi-fold change on the microarray results (Fig. 2E) and are known to be involved in EAE/MS pathogenesis [39, 40]. CD4^+^c-Met^+^ T cells expressed (i) higher level of CXCR3 and CCR6 compared to CD4^+^c-Met^−^ T cells in spleen, LN and CNS (Fig. 2G, top panels), (ii) higher level of CCR5 only in the CNS (Fig. 2G, middle and bottom panels), and (iii) whereas no difference was observed in CCR4 expression (Additional file 1: Fig. S1D).

### Phenotypic characterization of murine 2D2 CD4^+^Vα3.2^+^c-Met^+^ polarized Th1 T cells in vitro

**Fig. 3:**
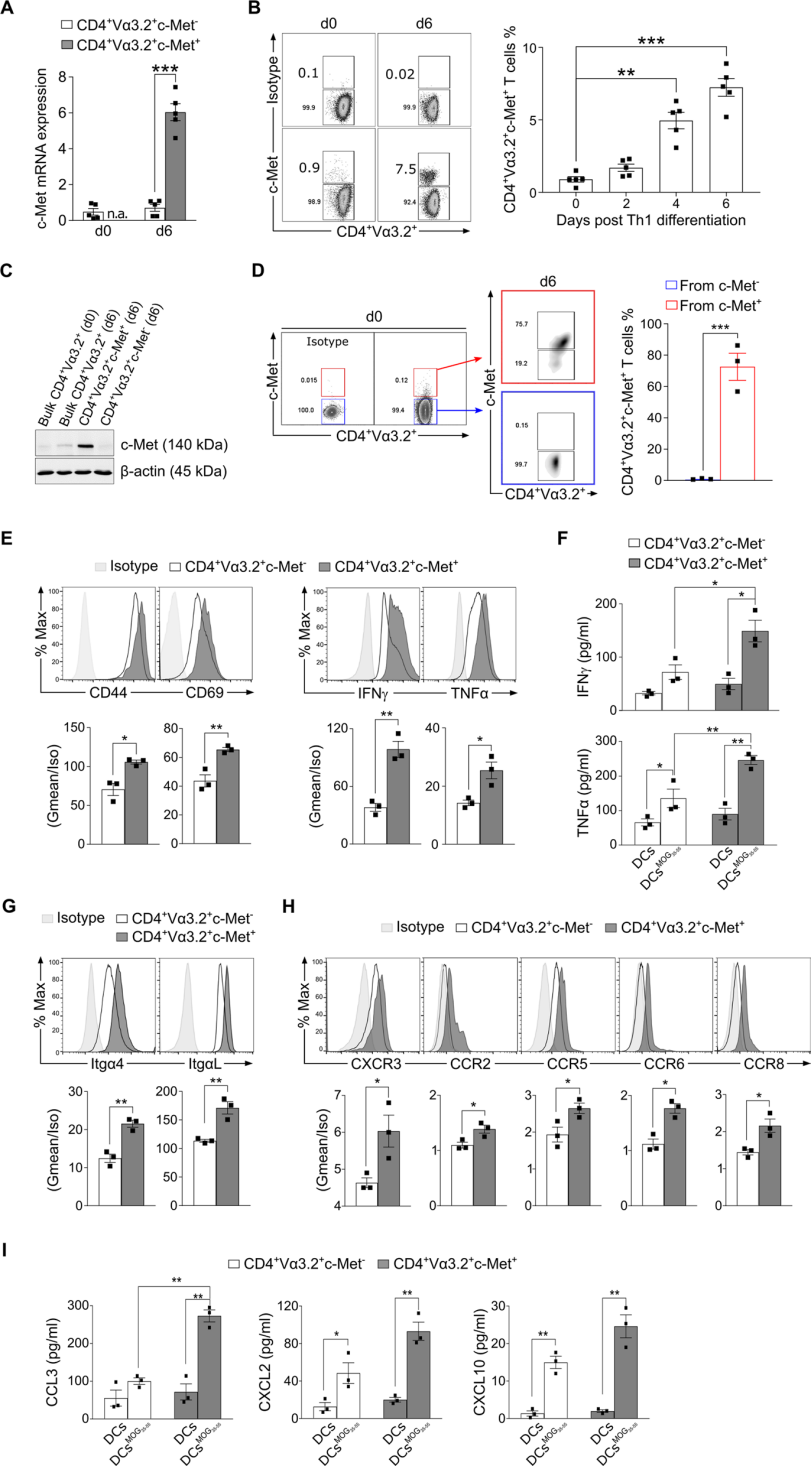
Phenotypic characterization of murine 2D2 CD4^+^Vα3.2^+^c-Met^+^ polarized Th1 T cells in vitro. **A** Relative expression of c‐Met mRNA by qRT-PCR in undifferentiated 2D2 CD4^+^Vα3.2^+^ T cells (d0), and FACS‐sorted CD4^+^Vα3.2^+^c‐Met^−^ and c‐Met^+^ T cells after in vitro polarization (d6) were assessed. **B** Representative flow cytometric plots that showed live 2D2 CD4^+^Vα3.2^+^ T cells (7AAD^−^CD45^+^CD4^+^, not shown) were analyzed during 6 days of in vitro Th1 differentiation (left panels), and mean percentages of c‐Met^+^ cells are shown (right panel). c-Met isotype control antibody (top panels) was used to define c-Met expression on 2D2 CD4^+^Vα3.2^+^ T lymphocytes. **C** Protein expression levels of c‐Met by Western blot in bulk 2D2 CD4^+^Vα3.2^+^ T cells undifferentiated (d0), versus Th1 differentiated (d6), and FACS‐sorted 2D2 CD4^+^Vα3.2^+^c‐Met^+^ and c‐Met^−^ T cells (d6). The expression of β-actin protein was assessed in all samples as control. Data are representative of three independent experiments. **D** Representative flow cytometric plots for the sorting of 2D2 CD4^+^Vα3.2^+^c-Met^−^ and CD4^+^Vα3.2^+^c-Met^+^ T cells before differentiation (d0, left panels) and after differentiation (d6, middle panels) of sorted CD4^+^Vα3.2^+^c-Met^−^ (blue) or CD4^+^Vα3.2^+^c-Met^+^ (red) T cells (middle panels). Quantification of CD4^+^Vα3.2^+^c-Met^+^ T cells proportion was shown on the right panel. **E** Representative flow cytometric histograms (top panels) and Gmean/isotype quantification (lower panel) of CD44, CD69, IFNγ and TNFα expression, gated on 2D2 CD4^+^Vα3.2^+^c‐Met^+^ and c‐Met^−^ T cells at day 6 post-Th1 differentiation. **F** Quantifications IFNγ and TNFα production by ELISA in supernatant from 2D2 CD4^+^ Vα3.2^+^c‐Met^−^ and c‐Met^+^ T cells, co-cultured for 48 h with MOG_35__–__55_-loaded DCs 6 day post-Th1 differentiation (triplicate). **G** Representative flow cytometric histograms (top panels) and Gmean/Isotype quantification (bottom panels) at day 6 post-Th1 differentiation of the expression of Itgα4 and ItgαL (VLA4 and LFA-1 integrins subunits, respectively) on 2D2 CD4^+^Vα3.2^+^c‐Met^−^ and c‐Met^+^ T cells. **H** Representative flow cytometric histograms (top panels) and Gmean/Isotype quantification (lower panel) at day 6 post-Th1 polarization of CXCR3, CCR2, CCR5, CCR6, and CCR8 chemokine receptors gated on 2D2 CD4^+^Vα3.2^+^c‐Met^−^ or c‐Met^+^ T cells. **I** Quantifications of CCL3, CXCL2 and CXCL10 production by multiplex assay in supernatant from 2D2 CD4^+^Vα3.2^+^c‐Met^−^ and c‐Met^+^ T cells, co-cultured for 48 h with MOG_35–55_-loaded DCs at day 6 post-Th1 differentiation (triplicate). Data are representative of three independent experiments and mean values ± SEM are shown; *p ≤ 0.05, **p ≤ 0.01 ***p ≤ 0.001, ****p ≤ 0.0001, unpaired two-tailed Student’s *t* test for two groups (**A**, **D**, **E**, **G**, **H**) or two-way ANOVA followed by Tukey’s post hoc test for multiple groups (**B**, **F**, **I**)

To assess c-Met expression on Th1 cells, we examined 2D2 TCR transgenic mice that express MOG_35–55_-specific Vα3.2 TCR. c-Met expression was assessed by qRT-PCR, flow cytometry and Western blot. A very low expression of c‐Met was detected in splenic resting CD4^+^Vα3.2^+^ T cells before MOG_35–55_-stimulation (d0), while a significant fraction of CD4^+^Vα3.2^+^ Th1 differentiated from 2D2 splenocytes post-MOG_35–55_-stimulation (d6) expresses c-Met (Fig. 3A–C). To understand the origin of c-Met expression on polarized Th1 CD4^+^Vα3.2^+^ cells, either CD4^+^Vα3.2^+^c-Met^+^ or c-Met^−^ T cells were isolated at day 0 from spleen cells and cultured under Th1 polarization conditions. After 6 days, c-Met expression was assessed by flow cytometry and remarkably, we noticed that CD4^+^Vα3.2^+^c-Met^−^ T cells were unable to generate c-Met^+^ cells, while all CD4^+^Vα3.2^+^c-Met^+^ cells were generated from pre-existing CD4^+^Vα3.2^+^c-Met^+^ cells (Fig. 3D). These results suggest that the increased population of c-Met^+^ found after polarization arise exclusively from the few c-Met^+^ naive cells that proliferate in vitro*.* Based on these results, we decided not to use of CD4 c-Met conditional knockout animals. Moreover, after Th1 polarization, CD4^+^Vα3.2^+^c-Met^+^ T cells expressed higher levels of activation markers, such as CD44 and CD69 (early activation marker) compared to CD4^+^Vα3.2^+^c-Met^−^ T cells (Fig. 3E, left panels). The same profile was observed for IFNγ and TNFα, two Th1 inflammatory mediators (Fig. 3E, right panels). To characterize the c-Met-expressing CD4^+^ T cells after MOG_35–55_-stimulation, FACS-sorted Th1 polarized 2D2 CD4^+^Vα3.2^+^c-Met^−^ and CD4^+^Vα3.2^+^c-Met^+^ cells were co-cultured with irradiated syngeneic DCs loaded with 20 μg/ml of MOG_35–55_ for 48 h. After re-stimulation with MOG_35–55_, we observed that CD4^+^Vα3.2^+^c-Met^+^ population secreted higher level of IFNγ and TNFα measured in the supernatant, compared to CD4^+^Vα3.2^+^c-Met^−^ T cells (Fig. 3F).

### 2D2 c-Met^+^ polarized Th1 T cells expressed higher level of integrins, chemokines and chemokine receptors

Since the data obtained by microarray analysis (Fig. 2) depicted an upregulation of several molecules related to adhesion and chemokines family, we investigated by flow cytometry the levels of adhesion molecules in 2D2 transgenic mice model following the gating strategy depicted in Additional file 1: Fig. S2A. Encephalitogenic CD4^+^ T cells were polarized under Th1 condition medium, in presence of MOG_35–55_ peptide. The results showed that CD4^+^Vα3.2^+^c-Met^+^ T cell population expressed higher level of Itgα4 and ItgαL than CD4^+^Vα3.2^+^c-Met^−^ T cells (Fig. 3G). However, we did not observe any difference in the expression of Itgα9 and PSGL-1 between the two populations (Additional file 1: Fig. S2B and C). These results are in line with both the microarray results and the pro-inflammatory profile of CD4^+^ T cells extracted from the CNS at EAE peak disease (Fig. 2). We next analyzed the expression levels of chemokine receptors involved in EAE pathogenesis and Th1 migration [41–44] including CXCR3, CCR2, CCR5, CCR6, and CCR8, on 2D2 Th1 c-Met^−^ and c-Met^+^, 6 days after Th1 differentiation in vitro. We found that all these chemokine receptors are upregulated in CD4^+^Vα3.2^+^c-Met^+^ compared to CD4^+^Vα3.2^+^c-Met^−^ T cells (Fig. 3H). By contrast, we did not observe any difference in the expression of CCR4 and CCR3 receptors between the two populations (Additional file 1: Fig. S2B). To measure the amount of chemokines produced by CD4^+^Vα3.2^+^ T cells upon antigen presentation, we performed an APC assay to quantify releasing molecules upon specific antigen stimulation. Polarized Th1 cells were co-cultured with irradiated MOG_35–55_-loaded DCs and analysis of the supernatant showed that CD4^+^Vα3.2^+^c-Met^+^ T cells were able to release higher amount of CCL3, CXCL2 and CXCL10 chemokines compared to CD4^+^Vα3.2^+^c-Met^−^ T cells (Fig. 3I).

### 2D2 CD4^+^Vα3.2^+^c-Met^+^ polarized Th1 T cells showed greater adhesion and transmigration properties in an endothelial adhesion assay

**Fig. 4:**
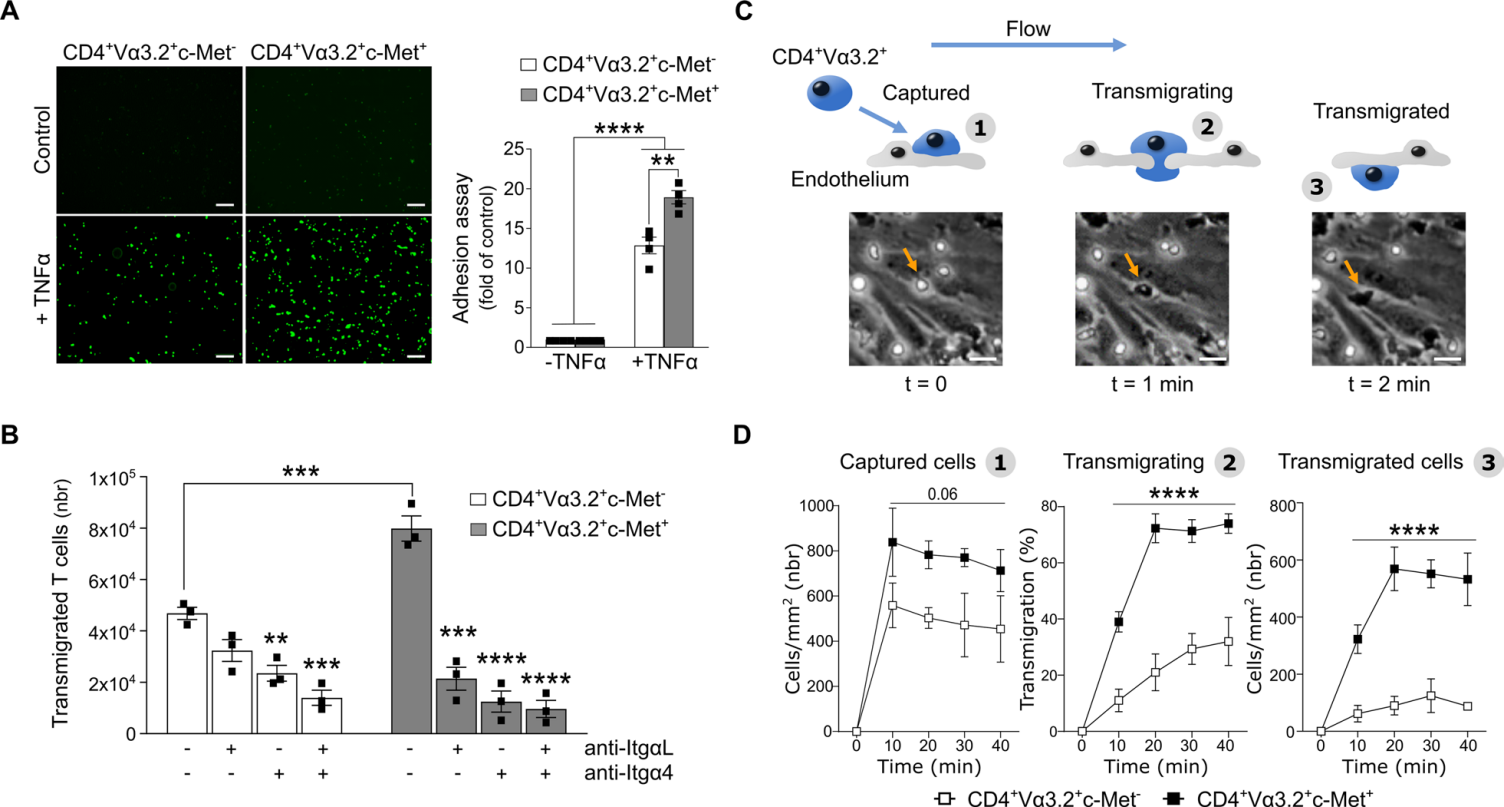
2D2 CD4^+^Vα3.2^+^c-Met^+^ polarized Th1 T cells showed greater adhesion and transmigration properties in vitro*.* **A** 2D2 CD4^+^Vα3.2^+^c-Met^−^ and c-Met^+^ T cells labeled with CFSE were seeded on the surface of confluent bEnd.3 cells. After 1 h of incubation and several washes, the attached 2D2 CD4^+^ T cells were analyzed. Representative immunofluorescent images (left panels) and quantification of the average numbers (right panel) of CFSE labeled T cells per well are shown. The values obtained in each group were calculated relative to the control condition (inactivated bEnd.3). Scale bar = 100 μm. **B** Transwell migration assays were performed by precoating the upper chambers with confluent monolayer of bEnd.3. A total of 10^5^ 2D2 CD4^+^Vα3.2^+^c-Met^−^ or c-Met^+^ T cells were blocked with 10ug/ml of indicated blocking antibodies, washed and seeded on the layer of activated bEnd.3. The transwell migration units were then incubated in presence of CXCL12 (100 ng/ml) in the lower chamber. After 6 h, all the cells contained in the lower chamber were harvested and the relative number of transmigrated cells was shown. **C** Schematic presentation of the flow assay system: T cell were captured from free flow and firmly adhered to HUVEC luminal surfaces (captured; step 1). T cell moves into the abluminal side by transmigrating between junctions of adjacent endothelial cells (transmigrating; step 2), which is followed by total transmigration (transmigrated T cells; step 3). Representative videomicrographs of CD4^+^Vα3.2^+^ Th1 cells (bottom panels) of the same area at indicated timepoint. Orange arrow indicates the same Th1 cell during the 3 phases; scale bar = 10 μm. **D** Adherent polarized 2D2 CD4^+^Vα3.2^+^c-Met^−^ and c-Met^+^ Th1 T cells on activated HUVECs were individually tracked and monitored for transmigration between different compartments for 50 min. Note that HUVEC cells were activated with CXCL12 and washed prior the adding of polarized Th1 cells. Captured, transmigrating and transmigrated T cells were analyzed separately. Data are presented as the means ± SD of 3 fields. Data are representative of three independent experiments and mean values ± SEM are shown (**A**, **B**); **p ≤ 0.01 ***p ≤ 0.001, ****p ≤ 0.0001, two-way ANOVA followed by Tukey’s post hoc test for multiple groups

Using a static endothelial adhesion assay, we compared the ability of CD4^+^Vα3.2^+^c-Met^−^ and CD4^+^Vα3.2^+^c-Met^+^ T cells to adhere on endothelial cells. While under resting conditions (inactivated endothelium) both cell populations were unable to adhere to the monolayer of mice brain derived endothelial (bEnd.3) cells. Specifically, CD4^+^Vα3.2^+^c-Met^+^ T cells showed a significant and robust adhesion capacity compared to CD4^+^Vα3.2^+^c-Met^−^ T cells on TNFα-activated endothelial cells (Fig. 4A). The capacity of CD4^+^Vα3.2^+^c-Met^+^ T cells to transmigrate through the monolayer of bEnd.3 cells was also significantly higher than CD4^+^Vα3.2^+^c-Met^−^ T cells in the presence of CXCL12 chemoattractant in the lower chamber. Noteworthy, the expression level of CXCR4 (CXCL12 receptor) is similar in both CD4^+^Vα3.2^+^c-Met^−^ and CD4^+^Vα3.2^+^c-Met^+^ cells (data not shown). The number of transmigrated cells were calculated by flow cytometry and normalized using counted beads as shown in Additional file 1: Fig. S3A.

Interestingly, when Itgα4 and ItgαL integrins were blocked separately or in combination, both c-Met^−^ and c-Met^+^ populations had an attenuated migratory ability, but the decrease was greater in CD4^+^Vα3.2^+^c-Met^+^. Compared to the untreated condition, the reduction of transmigrated cells was about 8 times lower in CD4^+^Vα3.2^+^c-Met^+^ T cells when treated with anti-Itgα4 alone (natalizumab) or Itgα4/ItgαL, while only a 2- and 3.4-fold decrease was observed in CD4^+^Vα3.2^+^c-Met^−^ T cells under the same conditions (Fig. 4B). Taken together, these results highlight the role of VLA4 subunit, Itgα4, to explain the increased pro-migratory profile in cell homing of CD4^+^Vα3.2^+^c-Met^+^ T cells.

### Increased trafficking profile of 2D2 CD4^+^Vα3.2^+^c-Met^+^ polarized Th1 T cells in a flow assay system

The higher adhesion and transmigration capacity of c-Met^+^ population prompted us to investigate how these cells position may vary in a system that mimics physiological aspects of inflammation using an innovative bio-imaging technology (MesenFlow Technologies). We monitored the interactions of individual T cells with activated HUVECs under fluid shear–stress conditions and recorded movement between luminal and abluminal sides every 30 s and compiled into a time-lapse short movie (Fig. 4C and Additional file 2: Video S1). Quantification of CD4^+^ T cells that change from a phase-white (captured) to a phase-black appearance (transmigrated) are expressed as a percentage of total T cells per unit field. We observed a significant increase of captured, transmigrating and transmigrated CD4^+^Vα3.2^+^c-Met^+^ T cells compared to CD4^+^Vα3.2^+^c-Met^−^ population within the observed 50 min time period (Fig. 4D). This profile is observed only under chronic inflammatory conditions, since in the absence of CXCL12 inflammatory chemokine (used to activate HUVEC cells and removed prior the adding of Th1 cells), c-Met^+^ and c-Met^−^ populations have similar trafficking profiles (Additional file 1: Fig. S3B).

### Adoptive transfer of 2D2 CD4^+^Vα3.2^+^c-Met^+^ Th1 cells increases EAE severity through Itgα4 overexpression

To evaluate the impact of Itgα4 on encephalitogenic Th1 in cell homing and EAE induction, we took advantage of transgenic 2D2 CD4^Cre^Itgα4^fl/fl^ mice that are deficient for Itgα4 (in > 98% of CD4^+^Vα3.2^+^ T cells). These mice are not deficient for Itgβ1—the second VLA4 subunit—or for LFA-1 integrin αL (Additional file 1: Fig. S4A). Itgα4-deficiency on CD4^+^Vα3.2^+^ Th1 cells did not influence the production of IFNγ (Additional file 1: Fig. S4B).

**Fig. 5:**
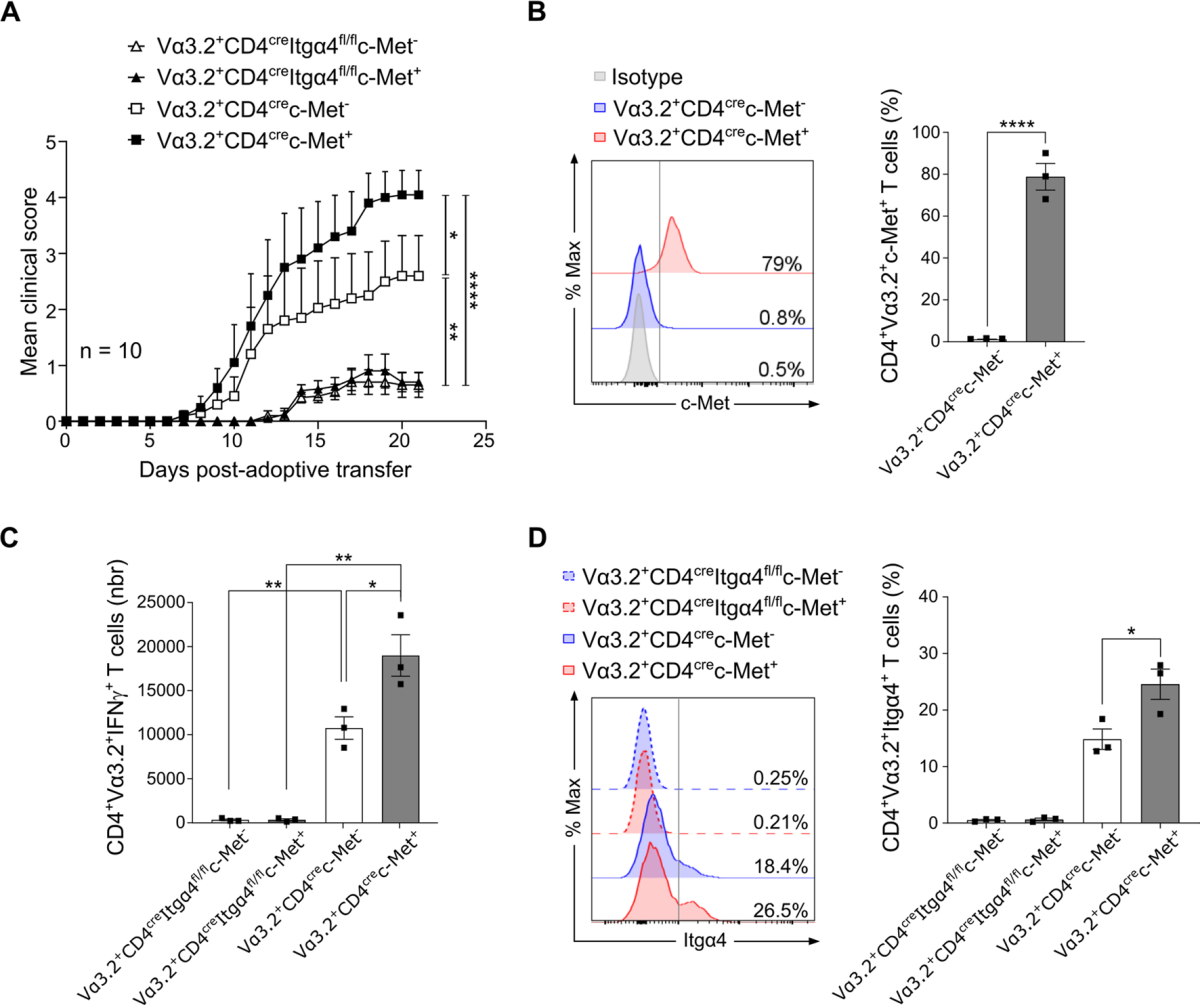
Adoptive transfer of 2D2 CD4^+^Vα3.2^+^c-Met^+^ Th1 cells induces more severe EAE through Itgα4 overexpression. **A** EAE was induced by i.p. transfer of 3 × 10^6^ 2D2 Vα3.2^+^CD4^Cre^c-Met^−^ or c-Met^+^ or 2D2 Vα3.2^+^CD4^Cre^Itgα4^fl/fl^ c-Met^−^ or c-Met^+^ Th1 followed by i.v. injection of PTX (67 ng/mouse) at d0 and d2. Data are representative of 2 independent experiments and mean clinical score were shown for n = 10 mice/group by two-way ANOVA with Bonferroni post Hoc test, *p < 0.05, **p < 0.01, ****p < 0.0001. **B** Representative flow cytometric histograms and quantification of c-Met-expressing CD4^+^Vα3.2^+^ infiltrating T cells at day 21 post-adoptive transfer. Data are representative of 2 independent experiments and mean values ± SEM were shown for n = 3 mice/group; ****p ≤ 0.0001 by unpaired two‐tailed Student's *t*‐test. **C** Quantification of CNS CD4^+^Vα3.2^+^IFNγ^+^ infiltrating T cells by flow cytometry at day 18 post-Th1 transfer. Data are representative of 2 independent experiments and mean values ± SEM for n = 3 mice/group are shown; *p ≤ 0.05, **p ≤ 0.01 by two-way ANOVA followed by Tukey’s post hoc test. **D** Representative flow cytometric histograms and quantification of Itgα4-expressing CD4^+^Vα3.2^+^ infiltrating T cells at day 18 post-adoptive transfer. Data are representative of 2 independent experiments and mean values ± SEM were shown for n = 3 mice/group; *p ≤ 0.05 by unpaired two‐tailed Student's *t*‐test

To assess the role of Itgα4 in EAE progression, we performed an adoptive transfer (AT) of Itgα4-deficient encephalitogenic CD4^+^Vα3.2^+^ T cells, expressing c-Met or not, generated from 2D2 CD4^Cre^Itgα4^fl/fl^ mice into C57BL/6J recipient mice. As expected, mice receiving Itgα4-deficient CD4^+^Vα3.2^+^ T cells by AT showed a delayed disease onset and a decreased EAE severity, regardless of c-Met expression (Fig. 5A). By contrast, the transfer of Itgα4-expressing CD4^+^Vα3.2^+^c-Met^+^ T cells induced a more severe EAE disease (Fig. 5A) with increased disease incidence and mortality (Additional file 1: Fig. S5A and B), when compared to the CD4^+^Vα3.2^+^c-Met^−^ T cells counterparts.

We next investigated the profile of CNS infiltrated T cells at AT peak disease (d18). As expected, Itgα4-deficient CD4^+^Vα3.2^+^ T cells—expressing c-Met or not—were barely detected in the CNS (Additional file 1: Fig. S5C), confirming the importance of Itgα4 on T cells migration into the CNS to induce EAE (Fig. 5A). We then analyzed c-Met expression stability of either c-Met^+^ or c-Met^−^ injected Th1 cells in the CNS at peak disease. An average of 20% of injected CD4^+^Vα3.2^+^c-Met^+^ lost the c-Met expression after 18 days of injection, while no c-Met expression was detected in the CNS of mice receiving CD4^+^Vα3.2^+^c-Met^−^ T cells (Fig. 5B), meaning that c-Met phenotype is maintained in vivo after AT. While the absolute number of total CD4^+^Vα3.2^+^c-Met^+^ T cells found in the CNS was not significantly higher than the CD4^+^Vα3.2^+^c-Met^−^ (Additional file 1: Fig. S5C), the absolute number of encephalitogenic IFNγ-producing T cells was increased in mice transferred with CD4^+^Vα3.2^+^c-Met^+^ T cells (Fig. 5C). Moreover, we found that CD4^+^Vα3.2^+^c-Met^+^ T cells—expressing Itgα4 or not—were producing more IFNγ compared to their CD4^+^Vα3.2^+^c-Met^−^ counterparts (Additional file 1: Fig. S5D). These results confirmed the pro-inflammatory profile of the c-Met^+^ population found in vitro (Fig. 3). Finally, in addition to their increase pro-inflammatory profile, infiltrated CD4^+^Vα3.2^+^c-Met^+^ T cells expressed significantly higher level of Itgα4 compared to their CD4^+^Vα3.2^+^c-Met^−^ counterpart (Fig. 5D), confirming that CD4^+^Vα3.2^+^c-Met^+^ T cells have a greater capacity to migrate into the CNS.

### Increased endogenous CD4^+^c‐Met^+^Itgα4^+^ T cells in MS patients

**Fig. 6:**
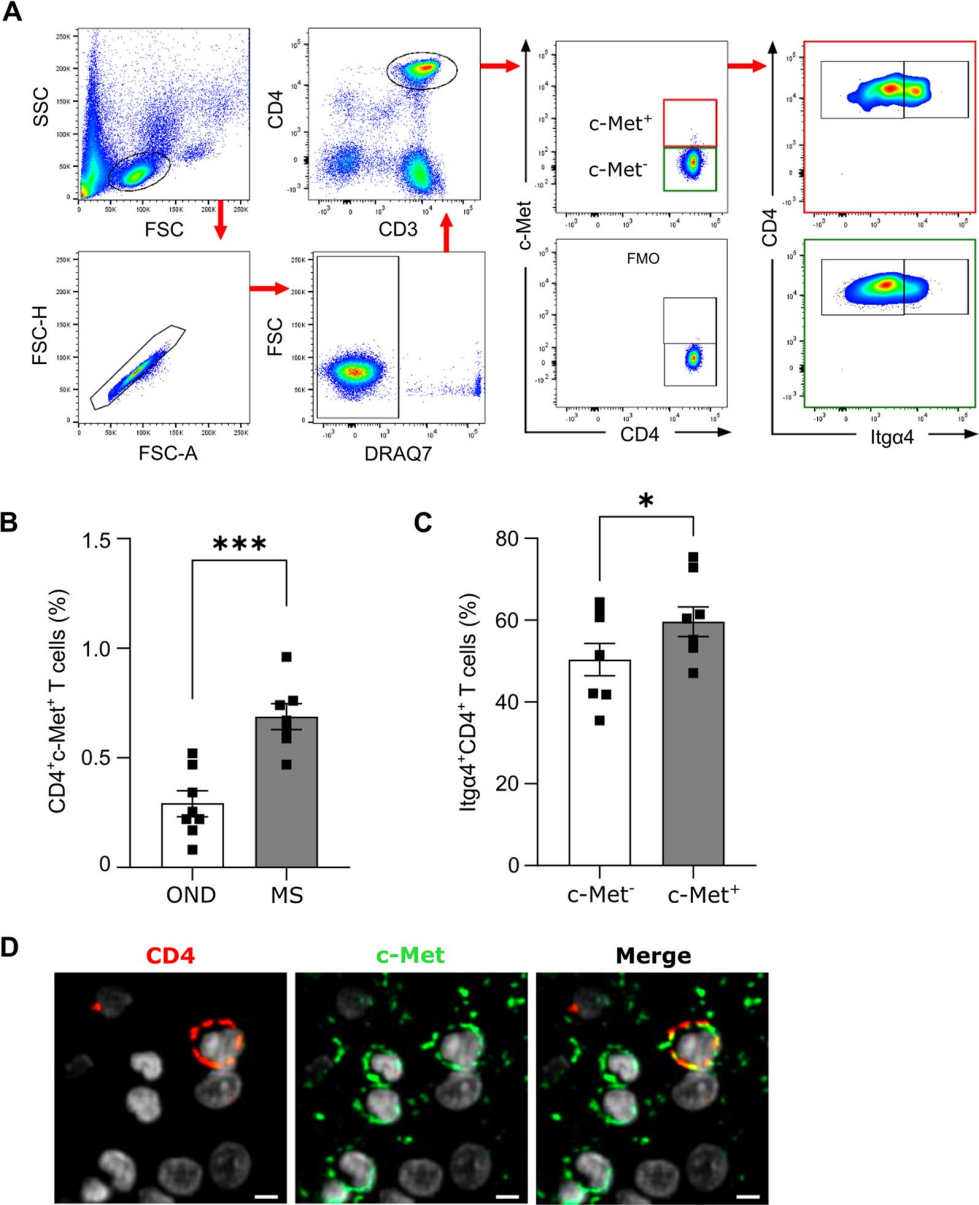
Detection of endogenous CD4^+^c‐Met^+^Itgα4^+^ T cells in MS patients. **A** Live lymphocytes from PBMCs of patients were first selected based on their morphology using FSC/SSC parameters, followed by the exclusion of doublets and dead cells (DRAQ7^+^). CD3^+^CD4^+^ T lymphocytes were subsequently selected and c-Met expression assessed on CD4^+^ T cells. **B** Flow cytometry quantification of circulating CD4^+^c-Met^+^ T cells in PBMCs from controls (other neurological disease (OND), n = 8) and multiple sclerosis (MS, n = 7) patients gated as described in A. Data are presented as mean ± SEM; ***p ≤ 0.001 by unpaired two‐tailed Student's *t*‐test. **C** Flow cytometry quantification of Itgα4^+^ on circulating CD4^+^c-Met^−^ and CD4^+^c-Met^+^ T cells in multiple sclerosis (MS, n = 7) patients. Data are presented as mean ± SEM; *p ≤ 0.05 by Wilcoxon matched-pairs signed rank test. **D** Representative immunofluorescent images of a brain biopsy from MS patient that showed the expression of CD4 (red), c-Met (green) and cell nuclei (white). Note that 22% of the CD4^+^ cells (50 ± 7) found in the section that measured around 6.2 mm^2^ (1000 μm × 6200 μm) were c-Met^+^ (11 ± 2). Scale bar = 5 μm

To investigate the clinical relevance of CD4^+^cMet^+^ T cells observed in animal models, we examined the expression of c-Met receptor and Itgα4 by CD4^+^ T cells in human PBMCs of matched-age untreated patients with MS and patients with other neurological disease (OND). We collected peripheral blood from these patients and assessed c-Met expression on circulating CD4^+^ T cells by flow cytometry following gating strategy (Fig. 6A). The results showed an increase of CD4^+^cMet^+^ population in the blood of MS patients (Fig. 6B) as previously described [25]. We next analyzed in MS patients the expression of Itgα4 on CD4^+^c‐Met^−^ and CD4^+^c‐Met^+^ T cells by flow cytometry and we found an increase expression of Itgα4 on CD4^+^c‐Met^+^ T cells, compared to their c-Met^−^ counterparts (Fig. 6C). We next wondered whether the CD4^+^c-Met^+^ T cells subset were present in the CNS from MS patients. By immunofluorescent staining for CD4 and c-Met, we were able to detect the presence of c-Met, not only on CD4^+^ T cells, but also on several cell population in MS brain. While it is already known that several CNS cell populations, including microglia, oligodendrocytes, astrocytes and neurons, and immune cells, such as dendritic and CD8^+^ T cells, are expressing c-Met [45–47], this is the first demonstration of the presence of CD4^+^c-Met^+^ T cells subset in the brain of MS patient (Fig. 6D).

Although further investigations on the function of these cells remain to be assessed in MS patients, these data may suggest a novel implication of the HGF/c-Met pathway in the development/maintenance of MS.

## Discussion

We recently demonstrated that a subgroup of encephalitogenic CD8^+^ T cell population expresses c-Met receptor [24]. The CD8^+^c-Met^+^ T cell population is significantly increased at peak disease and has a higher level of activation compared to CD8^+^c-Met^−^ T cells, similarly to a previous observation in a murine tumor model [30, 48]. Using ex vivo and in vitro strategies, we reported the capacity of HGF to reduce the encephalitogenic property of this subset of CTLs and suggest that expression of c-Met in CD8^+^ T cells may have a role in the causality of CNS pathology [24]. In addition, we uncovered the presence of human c-Met-expressing CD4^+^ T cells upon TCR triggering. CD4^+^c-Met^+^ T cells revealed an enhanced pro-inflammatory phenotype skewed towards a Th17 and Th17.1 polarization, and higher levels of Itgα4 compared to CD4^+^c-Met^−^ T cells [25]. Nevertheless, the expression of c-Met on CD4^+^ T cells—as well as its possible function—in model of neuroinflammation is unknown.

Here, we focused our study on c-Met expression by CD4^+^ T lymphocytes in EAE, a prototypical CD4^+^ Th1-mediated autoimmune disease. We found a notable increase of c-Met expression on CD4^+^ T lymphocytes during the acute phase of EAE. CD4^+^c-Met^+^ T cell population was characterized by a Th1 and Th17 profile, considered to be the major drivers of pathogenesis in both EAE and MS [5, 49–52]. Activation marker CD44 was highly expressed by CD4^+^c-Met^+^ T cell population. Interestingly, it is known that the expression of CD44 isoforms containing exon v6 is a prerequisite for c-Met activation by its ligand HGF in several tumor cells and in primary cells [53]. Investigating whether this new population CD4^+^c-Met^+^ is regulated by CD44v6 should require further investigations.

Inflammatory responses initiated by CD4^+^ T cells promotes tissue damage of the CNS in EAE and MS. Among the CD4^+^ T cells, Th1 cells and Th17 cells are dominant, the former producing IFNγ, the latter secreting IL-17, IL-21, IL-22, or GM-CSF [54, 55]. Once these cytokines are secreted in the CNS, they activate astrocytes and microglia, and recruit peripheral immune cells to inflammation sites [55]. Here, we demonstrated an increase of CD4^+^c-Met^+^ T cell population at EAE peak disease within the CNS. This population secretes greater amount of IFNγ, IL-17 and GM-CSF compared to their CD4^+^c-Met^−^ counterpart.

In vitro studies with Th1 polarized CD4^+^Vα3.2^+^c-Met^+^ T cells confirmed first that CD4^+^Vα3.2^+^c-Met^+^ subpopulation is generated from pre-existing proliferating cells and that their counterpart CD4^+^Vα3.2^+^c-Met^−^ subset is not the origin of this T cell population. The use of CD4 c-Met conditional knockout animals may help to understand the role of c-Met on CD4^+^ T cells in EAE, but since our results demonstrated that CD4^+^c-Met^−^ T cells were unable to generate CD4^+^c-Met^+^ T cells, we thus assumed that CD4^+^c-Met^−^ population is the proper control of their c-Met^+^ counterparts. In addition, using CD4^cre^c-Met^flox^ or inducible CD4^cre/ERT2^c-Met^flox^ mice will deplete c-Met not only in CD4^+^ T cells, but also in CD4^+^ monocytes and CD4^+^CD8^+^ T cells, which will be an important limiting factor to define the specific role of CD4^+^ T cells in our model. The inflammatory phenotype of the CD4^+^Vα3.2^+^c-Met^+^ T cells is characterized by an increase in the expression of both CD44 and CD69 activation markers, and augmented production and secretion of IFNγ. In adoptive transfer study, we have shown that in vitro polarized encephalitogenic 2D2 CD4^+^Vα3.2^+^ Th1 T cells expressing c-Met receptor are able to induce more severe EAE compared to their c-Met negative counterparts. Concerning the plasticity of c-Met expression, an average of 20% of Th1 cells lost this expression. However, 100% of c-Met^−^ Th1 injected cells maintained this phenotype at peak disease. Further investigations must be performed concerning the plasticity of CD4^+^c-Met^+^ T cells. These results suggest that c-Met could be an additional marker that designates a highly encephalitogenic T cell population.

We also observed that c-Met expression by CD4^+^ T cells in EAE was associated with increased expression of different chemokine receptors, involved in both Th1 (CXCR3, CCR5) and Th17 (CCR6) populations. We showed in vitro that c-Met signature on CD4^+^ T cells is associated with increased capacity to express higher level of chemokines CXCL2, CCL3 and CXCL10, known to be involved in EAE pathogenesis [56]. These data suggest an additional role of chemokines and their receptors in the pro-inflammatory functions of CD4^+^ T cells.

Numerous studies confirmed the predominant involvement of Itgα4—vascular cell adhesion molecule (VCAM)-1 subunit—in inflammatory cell recruitment into the CNS in different EAE models [57]. Indeed, Itgα4 contributes to rolling and mediate G-protein dependent arrest of endogenous encephalitogenic T cells [58]. The microarray study shows that gene expression of several adhesion molecules, including Itgα4, are overexpressed by CD4^+^c-Met^+^ T cells in the CNS at EAE peak disease, when compared to their CD4^+^c-Met^−^ counterparts. Itgα4 is known to play a significant role in EAE and MS pathogenesis [10–15]. The EAE results confirmed the overexpression of Itgα4 on CD4^+^c-Met^+^ T cell population at EAE peak disease, suggesting their high capacity to migrate into the CNS through the BBB during neuro-inflammatory conditions. This observation is confirmed by the demonstration of Itgα4 overexpression at the surface of CD4^+^c-Met^+^ T cells in in vitro model using 2D2 transgenic mice. Functional experiments using adhesion, transmigration and shear flow assays confirmed that both adhesion and transmigration functions are increased in CD4^+^c-Met^+^ T cells. This effect is inhibited when the cells are pre-treated with neutralizing anti-Itgα4 antibodies. In adoptive transfer studies with encephalitogenic Th1 cells, we pointed out the association between CD4^+^ T cells expressing both c-Met and Itgα4 and EAE severity. While the role of Itgα4 in EAE is known [10–15], here we demonstrate that, when associated with c-Met expression, the overexpression of Itgα4 worsen EAE severity. To understand the link between c-Met expression and Itgα4, and to determine the molecular underpinning, further studies are underway.

Our data indicate that a significant proportion of activated CD4^+^ T cells in EAE express c-Met receptor, while this expression remains negligible on naive CD4^+^ T cells, suggesting a correlation with pathological systemic inflammation. Non-stimulated 2D2 CD4^+^ Vα3.2^+^c-Met^−^ T cells polarization does not change c-Met receptor detection on the surface of activated cells, confirming that CD4^+^Vα3.2^+^c-Met^+^ T cell population is generated from pre-existing non-stimulated CD4^+^Vα3.2^+^c-Met^+^ T cells that proliferate after activation.

While the EAE data showed that both Th1 and Th17 T cell population are increased in the spleen, LN and CNS at peak disease, we have focused our in vitro and adoptive transfer investigations on Th1-polarized T cell population, because Itgα4 is specifically required for the homing of Th1 but not Th17 cells into the CNS [11]. Both Th1 and Th17 cells are capable of inducing EAE with different clinical outcomes, while Th1 cells mediate classic EAE with hind limb paralysis and Th17 cells induce ataxic gait in approximately half of the animals [59]. Within the CNS infiltrates, Th17 cells have been described as converting to a Th1 phenotype, but not vice versa [59].

In this study, we have also described an increase of CD4^+^c-Met^+^ T lymphocytes in the peripheral blood of MS patients and demonstrated that this population expressed higher level of Itgα4 compared to CD4^+^c-Met^−^ counterpart. This fraction of circulating CD4^+^c-Met^+^Itgα4^+^ T cells might play a key role in MS pathogenesis with pro-migratory function across the BBB. This theory is supported by the presence of this particular population that we found in the CNS of MS patients. A limitation of our study is due to the difficulty to obtain brain biopsies from MS patient. Further investigations are planned to better characterize this CD4^+^c-Met^+^ T cell population in MS pathogenesis, including its role in demyelination and axonal damage.

Unanswered questions arose from this study include whether c-Met is simply a marker of an activated phenotype or plays a causal role in the function of the c-Met^+^ population, which signalling pathways are involved in the generation of CD4^+^c‐Met^+^ T cells, what is the specific function of c-Met in the regulation of pro-inflammatory and pro-migratory CD4^+^ T cells, and how HGF regulates the functions of these cells. Further investigations to answer those questions should be addressed in the future.

## Conclusions

These results demonstrate that c-Met is a new signature of inflammatory and migratory encephalitogenic CD4^+^ T cells with the ability to transmigrate into the CNS. The receptor c-Met is a marker of activated CD4^+^ T cells expressing higher level of integrins, chemokines and chemokine receptors. Further investigations on the HGF/c-Met axis is mandatory to help the comprehension of EAE and MS pathogenesis, as well as to evaluating the development of new therapeutic approaches.

## Supplementary Information

**Additional file** **1**: **Figure S1. **MOG_35-55_-induced EAE clinical score. **(A) **MOG_35–55_-induced EAE in C57BL/6J mice by immunization with 200 μg of MOG_35–55_, emulsified in CFA on day 0. Mice also received 300ng of PTX i.v. on days 0 and 2. EAE disease severity was followed until chronic phase (d21) and mean clinical score ± SEM for n=10 mice/group were shown. **(B) **Percentage of disease incidence of immunized mice described in (A) is shown. **(C) **TNFα production by CD4^+^ T lymphocytes isolated at peak disease (d14) from spleen, LN and CNS after in vitro stimulation. Representative flow cytometric plots (left panels) and quantification (right panels) of CD4^+^c-Met^-^ vs c-Met^+^ T cells are depicted. Mean values ± SEM for n=8 mice/group are shown.** (D)** Representative flow cytometric histograms (left panels) and Gmean/Isotype quantification (right panels) of CCR4 and PSGL-1 expression on CD4^+^c-Met^-^ and c-Met^+^ T cells from spleen, LN and CNS extracted at peak disease (d14). Mean values ± SEM for n=5 mice/group are shown. **Figure S2. **2D2 CD4^+^Vα3.2^+^c-Met^-^ and c-Met^+^ polarized Th1 cells characterization in vitro. **(A)** Flow cytometry gating strategy for the identification of 2D2 CD4^+^Vα3.2^+^ T cells. Live lymphocytes were first selected for their morphology using FSC/SSC parameters, followed by the exclusion of doublets and dead cells (AQUA), and CD4^+^Vα3.2^+^ T lymphocytes were subsequently selected. **(B) **Representative flow cytometric histograms at day 6 post-Th1 differentiation of the expression of Itgα9 on 2D2 CD4^+^Vα3.2^+^c‐Met^-^ and c‐Met^+^ T cells. **(C)** Gmean/Isotype quantification by flow cytometry of 2D2 polarized Th1 cells at day 6 post-differentiation of CCR4, CCR3 and PSGL-1 gated on 2D2 CD4^+^Vα3.2^+^c‐Met^-^and c‐Met^+^ T cells. Data are representative of three independent experiments and mean values ± SEM for n=3 mice/group are shown. **Figure S3. **Trafficking profiles of 2D2 c-Met^+^ polarized Th1 T cells on non-activated HUVECs in an in vitro flow assay system.** (A)** Representative flow cytometry plots for the normalization of the relative number of transmigrated cells (red population) using fluorescent counting beads (blue population) upon stimulation with indicated antibodies. **(B)** Adherent polarized 2D2 CD4^+^Vα3.2^+^c-Met^-^ and c-Met^+^ Th1 T cells in coculture on non-activated HUVECs were individually tracked and monitored for transmigration between different compartments for 45 minutes. Captured (left panel), transmigrating (center panel) and transmigrated (right panel) T cells were analyzed separately. Data are presented as the means of 3 fields and are representative of three independents experiment. Mean values ± SD are shown.** (C) **Absolute numbers for c-Met^-^ and c-Met^+^ captured (top panels) and transmigrated (bottom panels) cells in presence (left panels) or absence (right panels) of CXCL12 at indicated time points counted during the in vitro flow assay system described in Fig. 4C and D. **Figure S4.** Integrin and cytokine expression of Th1 cells from 2D2 Vα3.2^+^CD4^Cre^Itgα4^fl/fl^ and Vα3.2^+^CD4^Cre^ control littermate mice. **(A)** Spleen cells from control 2D2 Vα3.2^+^CD4^Cre^ and 2D2 Vα3.2^+^CD4^Cre^Itgα4^fl/fl^ mice were polarized in vitro into Th1 cells and the expression of Itgα4, Itgb1 (VLA4 second associated subunit) and ItgαL (LFA-1 integrin subunit) were assessed by flow cytometry. Data arerepresentative of three independent experiments and mean values ± SEM for n=5 mice/group are shown; ***p≤0.001 by unpaired two‐tailed Student's *t*‐test. **(B)** Th1 polarized cells were stimulated with PMA/ionomycin and analyzed for cytokine secretion by intracellular cytokines staining. Percentages of IFNγ and IL-17 expressing Th1 cells from control 2D2 Vα3.2^+^CD4^Cre^ and 2D2 Vα3.2^+^CD4^Cre^Itgα4^fl/fl^ mice were determined. Data are representative of three independent experiments and mean values ± SEM for n=5 mice/group are shown. **Figure S5.** Adoptive transfer of Itgα4-deficient 2D2 CD4^+^c-Met^+^ Th1 cells induces delayed and reduced EAE incidence, severity, and decreased infiltrated T cell quantifications. **(A) **Percentage of disease incidence of EAE induced mice described in **Fig.5** are shown. **(B) **Survival curves of EAE induced mice described in **Fig. 5** are shown. **(C) **Quantification of CNS CD4^+^Vα3.2^+ ^infiltrating T cells by flow cytometry at day 18 post-Th1 transfer. Data are representative of 2 independent experiments and mean values ± SEM for n=3 mice/group are shown; **p≤0.01,***p≤0.001 by two-way ANOVA followed by Tukey’s post hoc test. **(D)** Representative flow cytometric plots of IFNγ and IL-17 production by Th1 infiltrating cells in the CNS at day 18 post-CD4^+^Vα3.2^+^ Th1 cells transfer and quantification of IFNγ-producing CD4^+^Vα3.2^+^ Th1 cells is shown on the right. Data are representative of 2 independent experiments and mean values ± SEM were shown for n=3 mice/group; *p≤0.05 by unpaired two‐tailed Student's *t*‐test. **Table S1.** Antibodies for flow cytometry

**Additional file 2: Video S1.** Bio-imaging analysis of shear flow assay. Time-lapse recording of CD4^+^Vα3.2^+^c-Met^-^ (left movie) and CD4^+^Vα3.2^+^c-Met^+^ (right movie) Th1 cells on activated HUVECs under flow for 50 min. Captured Th1 cells that subsequently transmigrate under the HUVEC monolayer switch from a phase-white to a phase-black appearance. (MP4 6270 KB)

## Data Availability

The data sets used and/or analyzed during the current study are available from the corresponding author on reasonable request.

## Abbreviations

MS: Multiple sclerosis
EAE: Experimental autoimmune encephalomyelitis
BBB: Blood brain barrier
CNS: Central nervous system
Th: T cell helper
c-Met: Mesenchymal epithelial transition factor
HGF: Hepatocyte growth factor
Itg: Integrin
IL: Interleukin
MOG: Myelin oligodendrocyte glycoprotein
CSF: Cerebrospinal fluid
PSGL: P-selectin glycoprotein ligand
VLA: Very late antigen
LFA: Leukocyte function-associated antigen
TCR: T cell receptor
OND: Other neurological disease
PBMC: Peripheral blood mononuclear cell
IFN: Interferon
LN: Lymph node
GM-CSF: Granulocyte-macrophage colony-stimulating factor
CTL: Cytotoxic T lymphocyte
DEG: Differentially expressed gene
DC: Dendritic cell
bEnd.3: Brain derived endothelial
AT: Adoptive transfer
VCAM: Vascular cell adhesion molecule
PTX: Pertussis toxin
