# Supplementary material for: CD4+c-Met+Itgα4+ T cell subset promotes murine neuroinflammation
Source: J Neuroinflammation. 2022 Apr 29;19:103. doi: 10.1186/s12974-022-02461-7 (PMC9052663; doi:10.1186/s12974-022-02461-7)
Supplement: Supplementary file 1 — Additional file 1: Figure S1. MOG35-55-induced EAE clinical score. (A) MOG35–55-induced EAE in C57BL/6J mice by immunization with 200 μg of MOG35–55, emulsified in CFA on day 0. Mice also received 300ng of PTX i.v. on days 0 and 2. EAE disease severity was followed until chronic phase (d21) and mean clinical score ± SEM for n=10 mice/group were shown. (B) Percentage of disease incidence of immunized mice described in (A) is shown. (C) TNFα production by CD4+ T lymphocytes isolated at peak disease (d14) from spleen, LN and CNS after in vitro stimulation. Representative flow cytometric plots (left panels) and quantification (right panels) of CD4+c-Met- vs c-Met+ T cells are depicted. Mean values ± SEM for n=8 mice/group are shown. (D) Representative flow cytometric histograms (left panels) and Gmean/Isotype quantification (right panels) of CCR4 and PSGL-1 expression on CD4+c-Met- and c-Met+ T cells from spleen, LN and CNS extracted at peak disease (d14). Mean values ± SEM for n=5 mice/group are shown. Figure S2. 2D2 CD4+Vα3.2+c-Met- and c-Met+ polarized Th1 cells characterization in vitro. (A) Flow cytometry gating strategy for the identification of 2D2 CD4+Vα3.2+ T cells. Live lymphocytes were first selected for their morphology using FSC/SSC parameters, followed by the exclusion of doublets and dead cells (AQUA), and CD4+Vα3.2+ T lymphocytes were subsequently selected. (B) Representative flow cytometric histograms at day 6 post-Th1 differentiation of the expression of Itgα9 on 2D2 CD4+Vα3.2+c‐Met- and c‐Met+ T cells. (C) Gmean/Isotype quantification by flow cytometry of 2D2 polarized Th1 cells at day 6 post-differentiation of CCR4, CCR3 and PSGL-1 gated on 2D2 CD4+Vα3.2+c‐Met-and c‐Met+ T cells. Data are representative of three independent experiments and mean values ± SEM for n=3 mice/group are shown. Figure S3. Trafficking profiles of 2D2 c-Met+ polarized Th1 T cells on non-activated HUVECs in an in vitro flow assay system. (A) Representative flow cytometry plots [file 12974_2022_2461_MOESM1_ESM.docx]

**SUPPLEMENTARY INFORMATION**

**CD4^+^c-Met^+^Itgα4^+^ T cell subset promotes murine neuroinflammation**

Mahdia Benkhoucha^1^, Ngoc Lan Tran^1^, Gautier Breville^1,2^, Isis Senoner^1^, Paul F. Bradfield^3^, Thalia Papayannopoulou^4^, Doron Merkler^5^, [Thomas Korn](https://pubmed.ncbi.nlm.nih.gov/?term=Korn+T&cauthor_id=24681001)^6,7,8^ and Patrice H. Lalive^1,2*^

^1^Department of Pathology and Immunology, Faculty of Medicine, University of Geneva, Geneva, Switzerland.

^2^Department of Neurosciences, Division of Neurology, University Hospital of Geneva, Geneva, Switzerland.

^3^MesenFlow Technologies SARL, Chemin des Aulx 14, Geneva, Switzerland.

^4^Division of Hematology, Department of Medicine, University of Washington, Seattle, WA

^5^Division of Clinical Pathology, Department of Pathology and Immunology, Faculty of Medicine, University of Geneva, Geneva, Switzerland.

^6^Department of Neurology, Klinikum rechts der Isar, Technical University of Munich, Munich, Germany.

^7^Institute for Experimental Neuroimmunology, Klinikum rechts der Isar, Technical University of Munich, Munich, Germany.

^8^Munich Cluster for Systems Neurology, SyNergy, Munich, Germany.

***Corresponding author:** Prof. Patrice H. Lalive, Phone: +41 22 372 83 18, Fax: +41 22 372 83 32, Email: patrice.lalive@hcuge.ch

**SUPPLEMENTARY FIGURES**

**
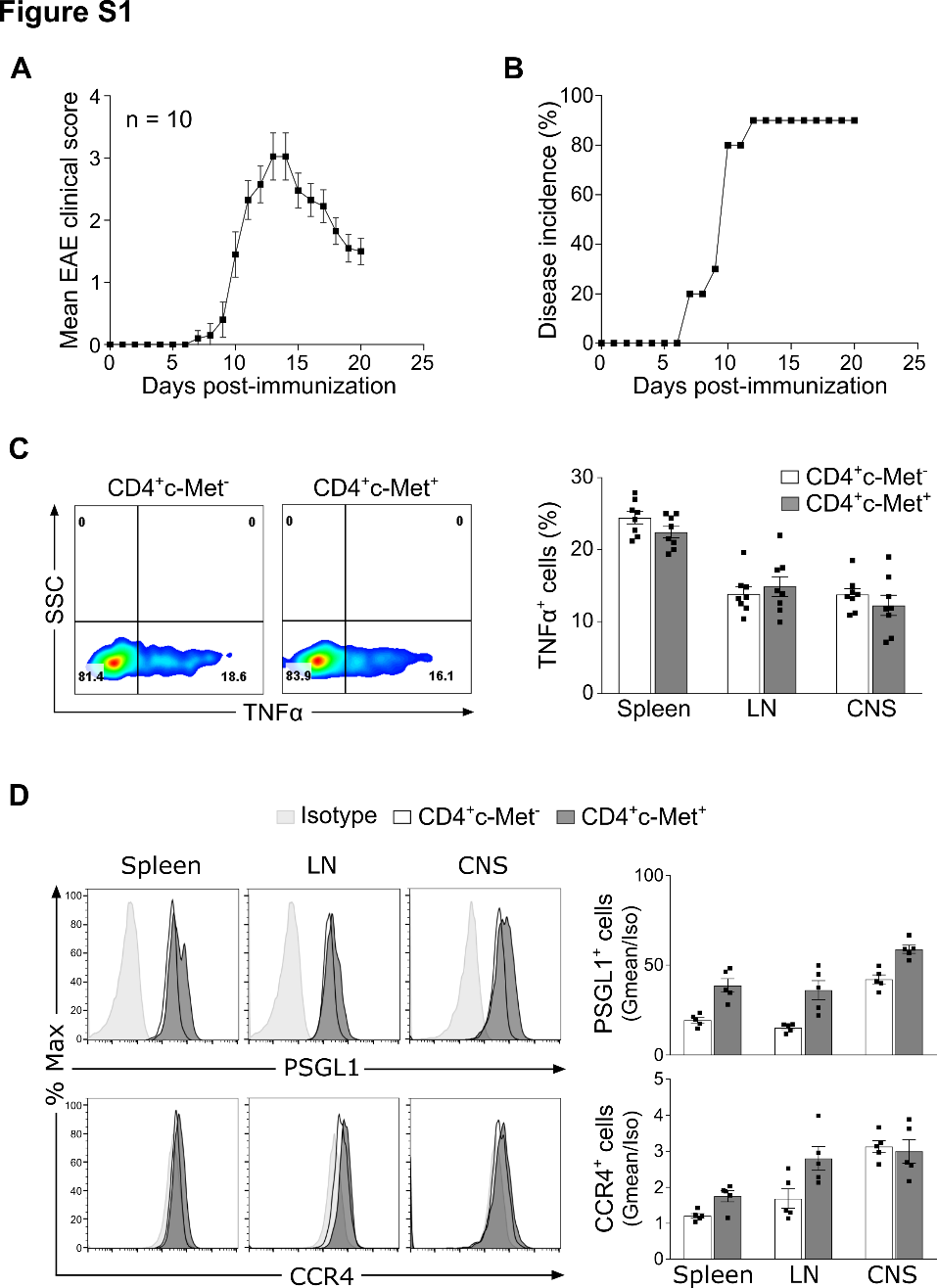
**

**Figure S1. MOG_35-55_-induced EAE clinical score**

**(A)** MOG_35–55_-induced EAE in C57BL/6J mice by immunization with 200μg of MOG_35–55_, emulsified in CFA on day 0. Mice also received 300ng of PTX i.v. on days 0 and 2. EAE disease severity was followed until chronic phase (d21) and mean clinical score ± SEM for n=10 mice/group were shown. **(B)** Percentage of disease incidence of immunized mice described in (A) is shown. **(C)** TNFα production by CD4^+^ T lymphocytes isolated at peak disease (d14) from spleen, LN and CNS after in vitro stimulation. Representative flow cytometric plots (left panels) and quantification (right panels) of CD4^+^c-Met^-^ vs c-Met^+^ T cells are depicted. Mean values ± SEM for n=8 mice/group are shown. **(D)** Representative flow cytometric histograms (left panels) and Gmean/isotype quantification (right panels) of CCR4 and PSGL1 expression on CD4^+^c-Met^-^ and c-Met^+^ T cells from spleen, LN and CNS extracted at peak disease (d14). Mean values ± SEM for n=5 mice/group are shown.

**
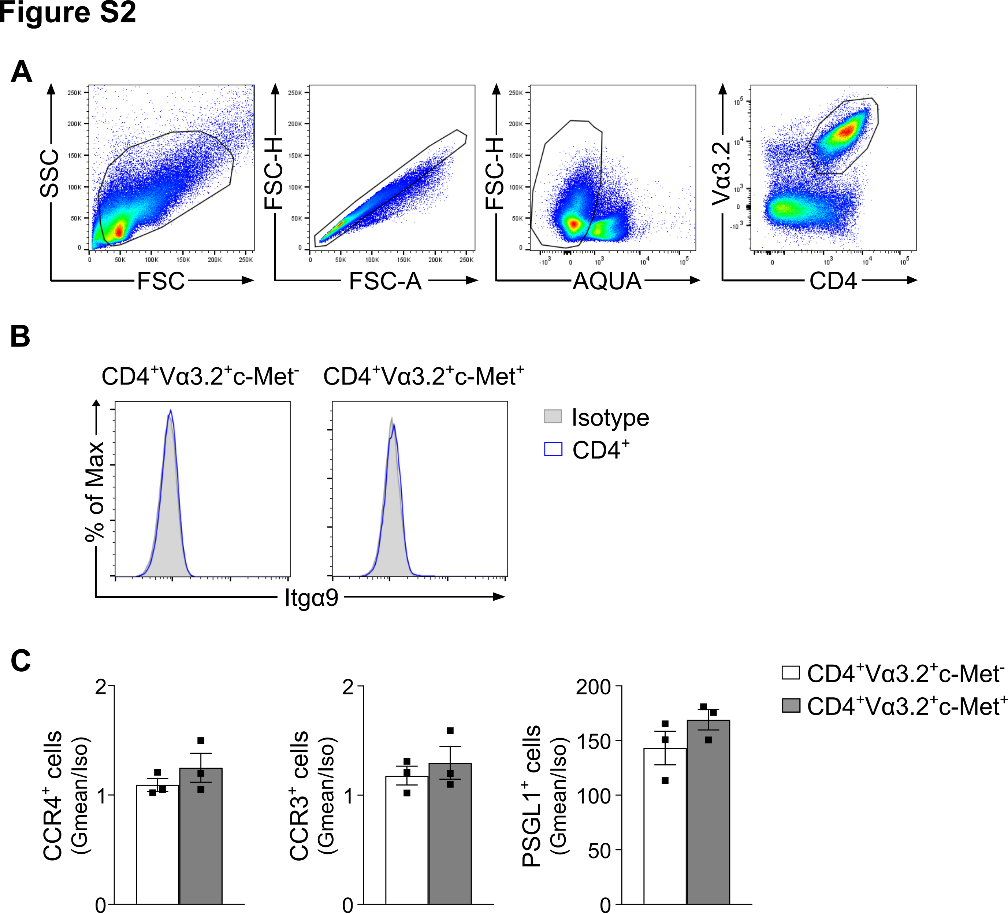
**

**Figure S2. 2D2 CD4^+^Vα3.2^+^c-Met^-^ and c-Met^+^ polarized Th1 cells characterization *in vitro***

**(A)** Flow cytometry gating strategy for the identification of 2D2 CD4^+^Vα3.2^+^ T cells. Live lymphocytes were first selected for their morphology using FSC/SSC parameters, followed by the exclusion of doublets and dead cells (AQUA), and CD4^+^Vα3.2^+^ T lymphocytes were subsequently selected. **(B)** Representative flow cytometric histograms at day 6 post-Th1 differentiation of the expression of Itgα9 on 2D2 CD4^+^Vα3.2^+^c‐Met^-^ and c‐Met^+^ T cells. **(C)** Gmean/isotype quantification by flow cytometry of 2D2 polarized Th1 cells at day 6 post-differentiation of CCR4, CCR3 and PSGL1 gated on 2D2 CD4^+^Vα3.2^+^c‐Met^-^ and c‐Met^+^ T cells. Data are representative of three independent experiments and mean values ± SEM for n=3 mice/group are shown.

**
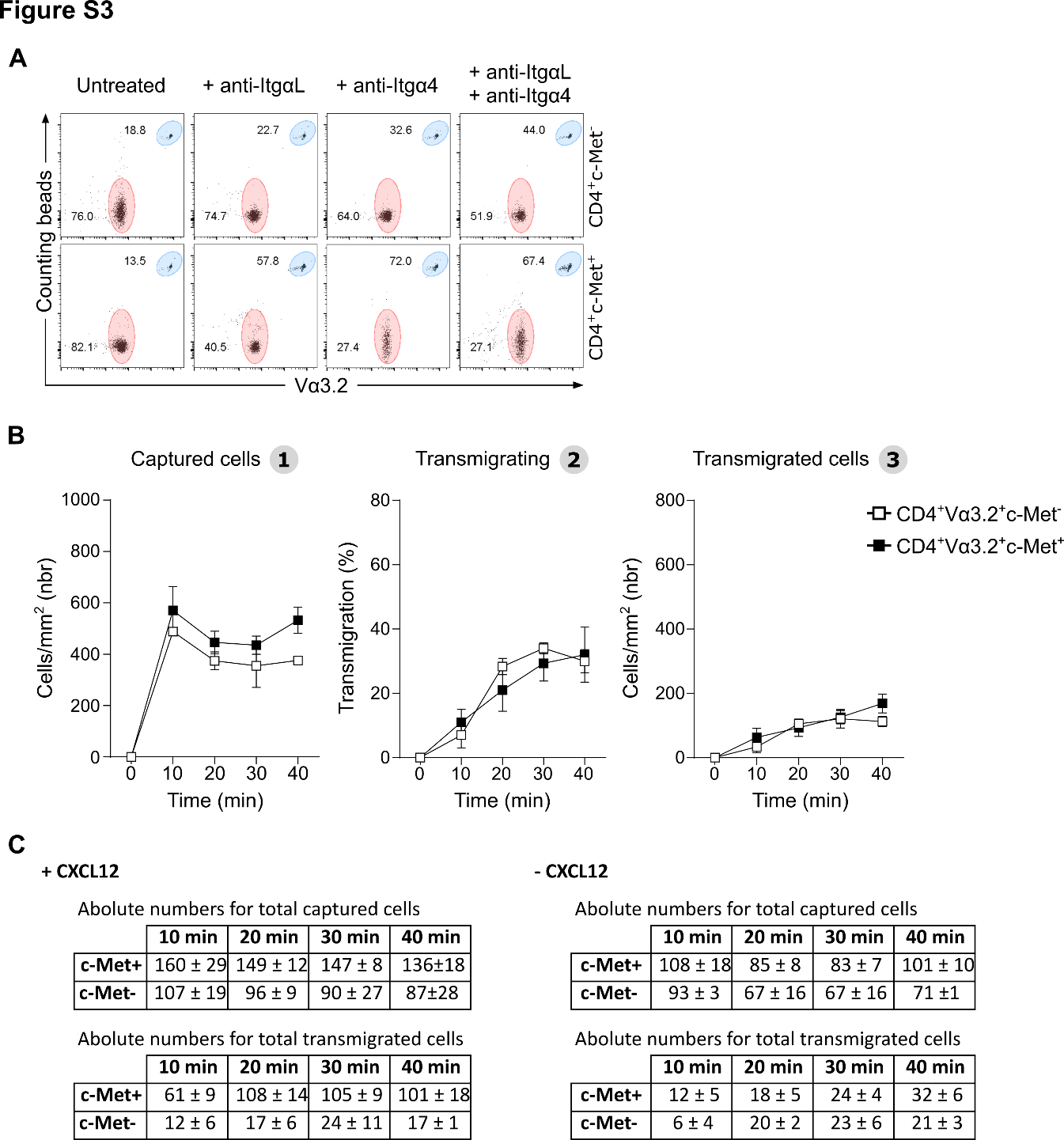
**

**Figure S3. Trafficking profiles of 2D2 c-Met^+^ polarized Th1 T cells on non-activated HUVECs in an *in vitro* flow assay system**

**(A)** Representative flow cytometry plots for the normalization of the relative number of transmigrated cells (red population) using fluorescent counting beads (blue population) upon stimulation with indicated antibodies. **(B)** Adherent polarized 2D2 CD4^+^Vα3.2^+^c-Met^-^ and c-Met^+^ Th1 T cells in coculture on non-activated HUVECs were individually tracked and monitored for transmigration between different compartments for 45 minutes. Captured (left panel), transmigrating (center panel) and transmigrated (right panel) T cells were analyzed separately. Data are presented as the means of 3 fields and are representative of three independents experiment. Mean values ± SD are shown. **(C)** Absolute numbers for c-Met^-^ and c-Met^+^ captured (top panels) and transmigrated (bottom panels) cells in presence (left panels) or absence (right panels) of CXCL12 at indicated time points counted during the *in vitro* flow assay system described in Fig. 4C and D.

**
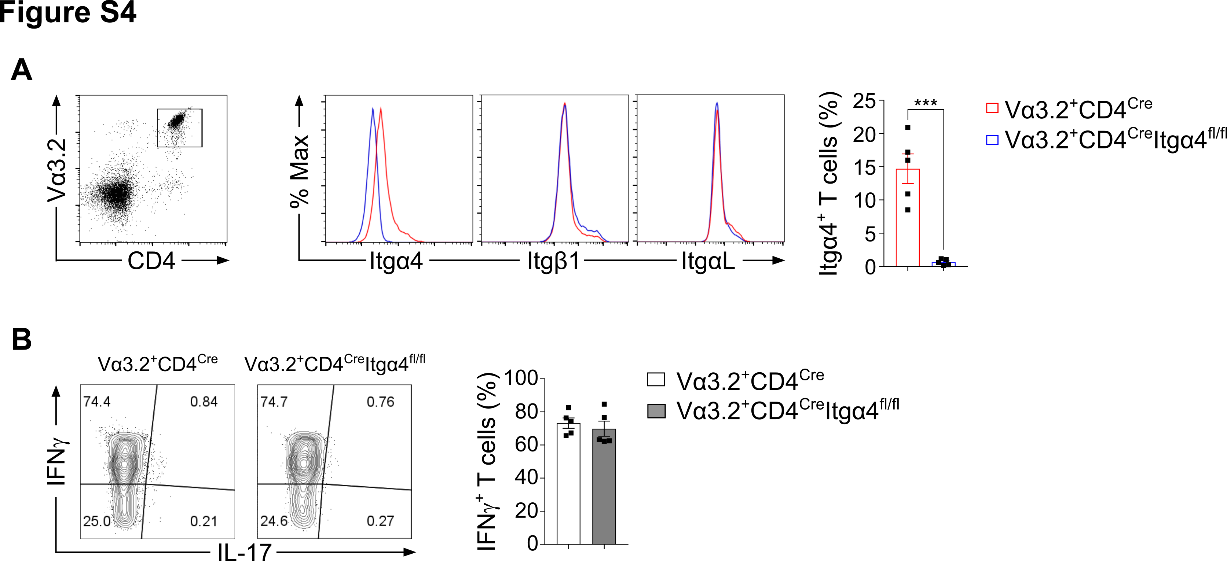
**

**Figure S4. Integrin and cytokine expression of Th1 cells from 2D2 Vα3.2^+^CD4^Cre^Itgα4^fl/fl^ and Vα3.2^+^CD4^Cre^ control littermate mice**

**(A)** Spleen cells from control 2D2 Vα3.2^+^CD4^Cre^ and 2D2 Vα3.2^+^CD4^Cre^Itgα4^fl/fl^ mice were polarized *in vitro* into Th1 cells and the expression of Itgα4, Itgβ1 (VLA4 second associated subunit) and ItgαL (LFA-1 integrin subunit) were assessed by flow cytometry. Data are representative of three independent experiments and mean values ± SEM for n=5 mice/group are shown; ***p≤0.001 by unpaired two‐tailed Student's t‐test. **(B)** Th1 polarized cells were stimulated with PMA/ionomycin and analyzed for cytokine secretion by intracellular cytokines staining. Percentages of IFNγ and IL-17 expressing Th1 cells from control 2D2 Vα3.2^+^CD4^Cre^ and 2D2 Vα3.2^+^CD4^Cre^Itgα4^fl/fl^ mice were determined. Data are representative of three independent experiments and mean values ± SEM for n=5 mice/group are shown.

**
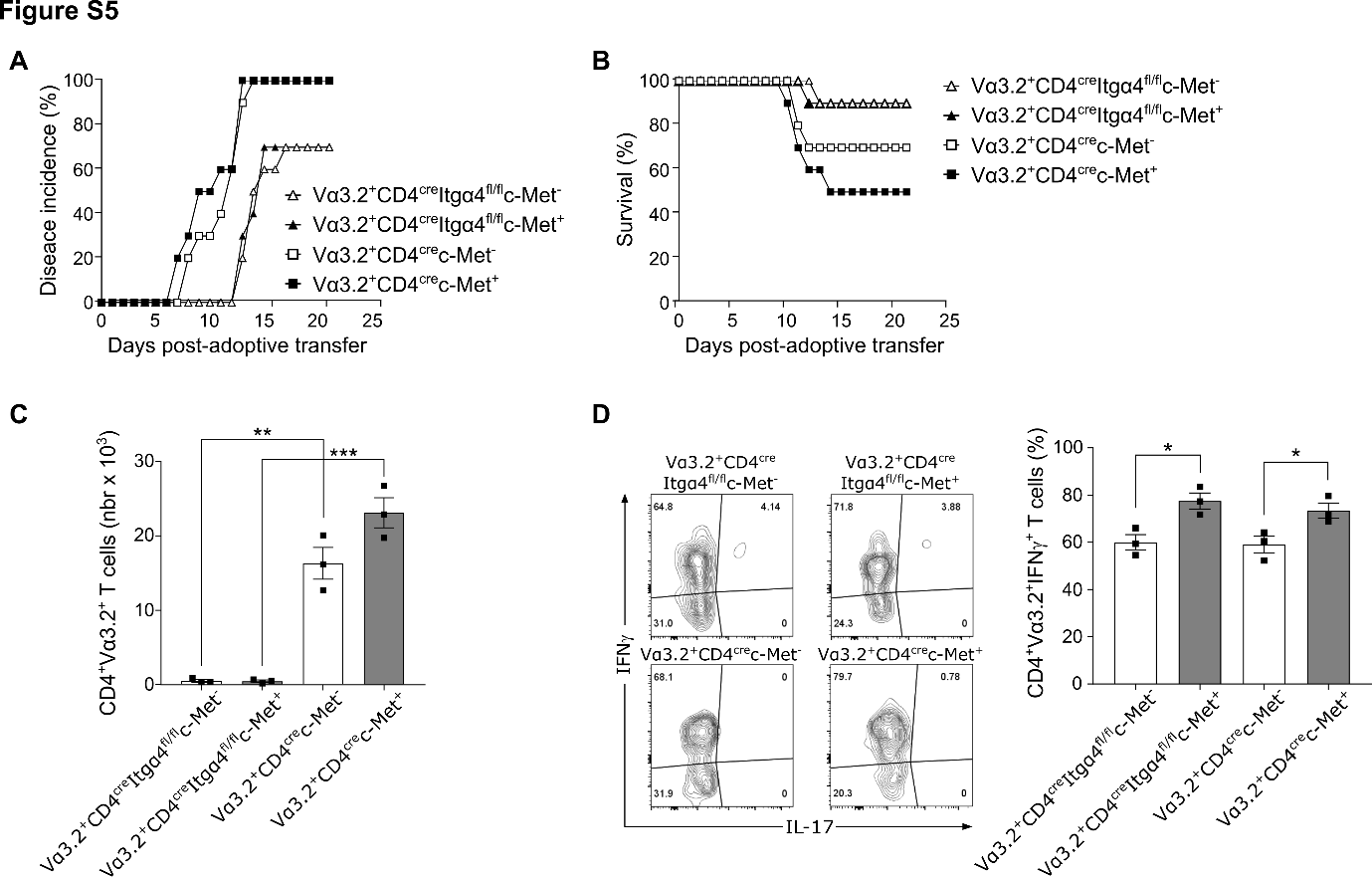
**

**Figure S5. Adoptive transfer of Itgα4-deficient 2D2 CD4^+^c-Met^+^ Th1 cells induces delayed and reduced EAE incidence, severity, and decreased infiltrated T cell quantifications**

**(A)** Percentage of disease incidence of EAE induced mice described in **Fig. 5** are shown. **(B)** Survival curves of EAE induced mice described in **Fig. 5** are shown. **(C)** Quantification of CNS CD4^+^Vα3.2^+^ infiltrating T cells by flow cytometry at day 18 post-Th1 transfer. Data are representative of 2 independent experiments and mean values ± SEM for n=3 mice/group are shown; **p≤0.01, ***p≤0.001 by two-way ANOVA followed by Tukey’s post hoc test. **(D)** Representative flow cytometric plots of IFNγ and IL-17 production by Th1 infiltrating cells in the CNS at day 18 post-CD4^+^Vα3.2^+^ Th1 cells transfer and quantification of IFNγ-producing CD4^+^Vα3.2^+^ Th1 cells is shown on the right. Data are representative of 2 independent experiments and mean values ± SEM were shown for n=3 mice/group; *p≤0.05 by unpaired two‐tailed Student's t‐test.

**SUPPLEMENTARY TABLE**

**Table S1: Antibodies for flow cytometry**

| **Antibodies** | **Species reactivity** | **Clone** | **Fluorochrome** | **Supplier** | **RRID number** |
| --- | --- | --- | --- | --- | --- |
| **CD45** | **Anti-mouse** | **30-F11** | **APC-Cy7** | **BD Pharmingen** | **AB_394606** |
| **CD4** | **Anti-mouse** | **RM4-5** | **PercP-Cy5.5** | **eBioscience** | **AB_467067** |
| **Vα3.2** | **Anti-mouse** | **RR3-16** | **APC, PercP-ef710** | **eBioscience** | **AB_10854272** |
| **CD3** | **Anti-mouse** | **17A2** | **BV605** | **BD Horizon** | **AB_2732063** |
| **c-Met** | **Anti-mouse** | **eBioclone7** | **FITC** | **Invitrogen** | **AB_494112** |
| **CD44** | **Anti-mouse** | **IM7** | **BV605, BUV737** | **BD Horizon** | **AB_2870126** |
| **CD69** | **Anti-mouse** | **H1.2F3** | **BUV 395** | **BD Bioscience** | **AB_2739968** |
| **ItgαL** | **Anti-mouse** | **M17/4** | **APC** | **Invitrogen** | **AB_11217471** |
| **Itgα4** | **Anti-mouse** | **R1-2** | **Pe-Cy7** | **Invitrogen** | **AB_2744779** |
| **Itgα9** | **Anti-mouse** | **Polyclonal** | **PE** | **Invitrogen** | **AB_2610554** |
| **Itgβ1** | **Anti-mouse** | **HMβ1-1** | **PE** | **Biolegend** | **AB_312884** |
| **PSGL1** | **Anti-mouse** | **2PH1** | **BV421** | **BD Horizon** | **AB_2737808** |
| **CXCR3** | **Anti-mouse** | **CXCR3-173** | **PE-ef610** | **Invitrogen** | **AB_2802385** |
| **CCR2** | **Anti-mouse** | **SA203G11** | **BV650** | **Biolegend** | **AB_2721553** |
| **CCR4** | **Anti-mouse** | **2G12** | **PE** | **Biolegend** | **AB_1236367** |
| **CCR5** | **Anti-mouse** | **HM-CCR5** | **APC, PE-Cy7** | **Biolegend** | **AB_2617013** |
| **CCR6** | **Anti-mouse** | **29-2L17** | **BV421** | **Biolegend** | **AB_2715923** |
| **CCR8** | **Anti-mouse** | **SA214G2** | **PE** | **Biolegend** | **AB_2617032** |
| **CCR3** | **Anti-mouse** | [**J073E5**](https://www.biolegend.com/en-us/search-results?Clone=J073E5) | **PE-Cy7** | **Biolegend** | [**AB_2565739**](http://antibodyregistry.org/AB_2565739) |
| **INFγ** | **Anti-mouse** | **XMG1.2** | **APC** | **Invitrogen** | **AB_2688063** |
| **TNFα** | **Anti-mouse** | **MP6-XT22** | **PE** | **BD Pharmingen** | **AB_395380** |
| **IL-17** | **Anti-mouse** | **eBio17B7** | **PercP-Cy5.5** | **eBioscience** | **AB_10732356** |
| **GM-CSF** | **Anti-mouse** | **MP1-22E9** | **BV421** | **BD Bioscience** | **AB_2738929** |
| **CD3** | **Anti-human** | **UCHT1** | **APC-Cy7** | **Biolegend** | **AB_893299** |
| **CD4** | **Anti-human** | **OKT4** | **PE-Cy7** | **Biolegend** | **AB_571959** |
| **CD49d/Itgα4** | **Anti-human** | **9F10** | **BV711** | **Biolegend** | **AB_2687198** |

**SUPPLEMENTARY VIDEO**

**Video S1. Bio-imaging analysis of shear flow assay**

Time-lapse recording of CD4^+^Vα3.2^+^c-Met^-^ (left movie) and CD4^+^Vα3.2^+^c-Met^+^ (right movie) Th1 cells on activated HUVECs under flow for 50 min. Captured Th1 cells that subsequently transmigrate under the HUVEC monolayer switch from a phase-white to a phase-black appearance.
